# Modeling ERBB receptor-regulated G1/S transition to find novel targets for *de novo *trastuzumab resistance

**DOI:** 10.1186/1752-0509-3-1

**Published:** 2009-01-01

**Authors:** Özgür Sahin, Holger Fröhlich, Christian Löbke, Ulrike Korf, Sara Burmester, Meher Majety, Jens Mattern, Ingo Schupp, Claudine Chaouiya, Denis Thieffry, Annemarie Poustka, Stefan Wiemann, Tim Beissbarth, Dorit Arlt

**Affiliations:** 1 Division of Molecular Genome Analysis, German Cancer Research Center, Im Neuenheimer Feld 580, 69120 Heidelberg, Germany; 2 Current address: Phadia GmbH, Munzinger Strasse 7, 79010 Freiburg, Germany; 3 Current address: Roche Diagnostics GmbH, Nonnenwald 2, 82377 Penzberg, Germany; 4 Technologies Avancées pour le Génome et la Clinique, INSERM U928, Université de la Méditerranée, Campus Scientifique de Luminy – Case 928, 13288 Marseille, France

## Abstract

**Background:**

In breast cancer, overexpression of the transmembrane tyrosine kinase ERBB2 is an adverse prognostic marker, and occurs in almost 30% of the patients. For therapeutic intervention, ERBB2 is targeted by monoclonal antibody trastuzumab in adjuvant settings; however, *de novo *resistance to this antibody is still a serious issue, requiring the identification of additional targets to overcome resistance. In this study, we have combined computational simulations, experimental testing of simulation results, and finally reverse engineering of a protein interaction network to define potential therapeutic strategies for *de novo *trastuzumab resistant breast cancer.

**Results:**

First, we employed Boolean logic to model regulatory interactions and simulated single and multiple protein loss-of-functions. Then, our simulation results were tested experimentally by producing single and double knockdowns of the network components and measuring their effects on G1/S transition during cell cycle progression. Combinatorial targeting of ERBB2 and EGFR did not affect the response to trastuzumab in *de novo *resistant cells, which might be due to decoupling of receptor activation and cell cycle progression. Furthermore, examination of c-MYC in resistant as well as in sensitive cell lines, using a specific chemical inhibitor of c-MYC (alone or in combination with trastuzumab), demonstrated that both trastuzumab sensitive and resistant cells responded to c-MYC perturbation.

**Conclusion:**

In this study, we connected ERBB signaling with G1/S transition of the cell cycle *via *two major cell signaling pathways and two key transcription factors, to model an interaction network that allows for the identification of novel targets in the treatment of trastuzumab resistant breast cancer. Applying this new strategy, we found that, in contrast to trastuzumab sensitive breast cancer cells, combinatorial targeting of ERBB receptors or of key signaling intermediates does not have potential for treatment of *de novo *trastuzumab resistant cells. Instead, c-MYC was identified as a novel potential target protein in breast cancer cells.

## Background

Anticancer drugs which are in clinical use show their effect by acting as non-selective anti-proliferative agents which kill also the proliferating normal cells in the tumor microenvironment [1]. The past few decades witnessed the development of targeted therapies including monoclonal antibodies, which aim at targeting certain antigens expressed on the surface of cancer cells with high specificity. In particular, adding trastuzumab, a recombinant humanized monoclonal antibody directed against the ectodomain of the receptor tyrosine kinase ERBB2, to regimens containing existing chemotherapeutic agents has significantly improved clinical outcomes for breast cancer patients. However, *de novo *and acquired resistance to targeted therapeutics are common and the next challenges for the contemporary cancer researchers [2].

The ERBB family of receptor tyrosine kinases is composed of four receptors that have the ability to form homo- and heterodimers, and couple binding of extracellular growth factors to intracellular signal transduction pathways [3,4]. ERBB2, the main player of the ERBB network, does not show any ligand binding activity, but has high dimerization affinity [5,6]. The abnormal activation of ERBB receptors through gene amplification, mutations, or protein overexpression has been linked to breast cancer prognosis [7]. Trastuzumab is administrated to ERBB2-overexpressing breast cancer patients [8,9]. The drug shows its effect by inducing antibody-dependent cellular cytotoxicity (ADCC), disrupting the downstream signaling of ERBB2 and also resulting in G1/S cell cycle arrest [10]. However, the response rate to trastuzumab is rather low, with a range from 12% to 34% having been reported for a median duration of 9 months [11,12]. Hence, at least two third of the patients are *de novo *resistant. On the cellular level, this might be caused by cancer cells being able to overcome cell cycle arrest despite targeting the ERBB2 receptor. Therefore, additional targets have to be identified, which should avoid bypass of cell cycle arrest mechanisms.

The cell cycle of eukaryotic organisms is tightly regulated by the cyclin-dependent kinases (CDKs) and their activation partners, cyclins [13], which lead cells through the well-ordered G1-, S-, G2-, and M-phases. It has been shown that ERBB2 regulates G1/S transition during cell cycle progression by modulating the activity of the Cyclin D, Cyclin E/CDK complex, the c-MYC oncogene, and the p27 kinase inhibitor [7,14]. The restriction points within different cell cycle phases represent key checkpoints, where the critical decisions are made for the cells to divide. At the G1/S restriction point of the cell cycle, cells are committed to enter S phase where DNA replication takes place [15]. This process is regulated by Cyclin D/CDK4/6 and Cyclin E/CDK2 complexes, which phosphorylate and thereby inactivate tumor suppressor retinoblastoma protein pRB [16-18]. Hyperphosphorylation of pRB results in the release of the E2F transcription factor that then initiates the transcription of essential genes for DNA replication [19]. In both normal and tumor cells, pRB oscillates between an active (hypophosphorylated) state in early G1 and an inactive (hyperphosphorylated) state in the late G1, S and G2/M phases [18]. Therefore, phosphorylation and subsequent inactivation of pRB represents a key event governing cell proliferation.

There have been few studies which applied systems biology approaches to identify novel markers [20] and to define drug target networks in human cancer and other pathologies [21]. In this study, we focused on the regulation of pRB through ERBB-receptor signaling at a network level in a *de novo *trastuzumab resistant cell system to identify new potential perturbation points leading to cell cycle arrest. Instead of single candidate gene approach, which generally examines the role of a single protein considering it either in conjunction with a second protein or regardless to other proteins, we applied a systems biology approach to identify the role of each component in the context of protein interaction networks. This strategy is motivated by the fact that cells react to perturbation of a single protein by taking advantage of using alternative ways to keep the system robust. In drug resistance, these alternative ways allow bypassing the inhibitory effect of drug treatment. Therefore, in order to find the uncommon perturbations to which cells cannot find an efficient way to react, we first integrated published data to build the protein network for ERBB-receptor regulated cell cycle progression, then combined qualitative dynamical modeling and robust experimental approaches, and finally predicted suitable and efficient targets for individual or combinatorial treatments in *de novo *trastuzumab resistance in breast cancer as a model system.

We used the Boolean logical framework for the dynamical modeling and analysis of the biological network. This framework simplifies the regulatory activity of proteins by considering them as all or none devices. More precisely, each protein is defined as being either active (value 1) or inactive (value 0) depending on its abundance or activity level. We selected 18 proteins connecting ERBB receptor signaling to the G1/S transition of cell cycle, and defined logical rules to describe their regulations with regard to literature information. Modeling and loss of function simulations of the network proteins were performed using the modeling and simulation software GINsim [22,23].

Experimental perturbations of each network element using RNAi and following measurements of their effects on the output protein allowed us to compare simulations with the experimental results. Quantitative measurements of protein abundance and activation states using reverse phase protein arrays (RPPA) [24,25] enabled us to reverse engineer the interactions of proteins in the cell system we used, and to compare the experimental network data with published results of single protein analysis. Utilizing specific inhibitors against potential targets alone or in combination with trastuzumab, we further validated the RNAi experiments and finally defined potential future therapeutic strategies.

## Results

### Characterization of the *de novo *trastuzumab resistant cell system

**Figure 1:**
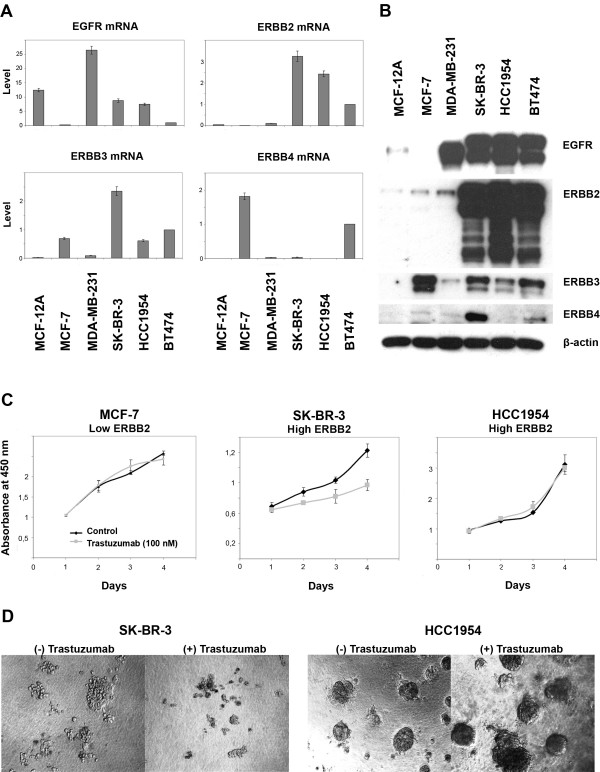
**Characterization of breast cell lines for trastuzumab resistance/sensitivity**. A. qRT-PCR to determine ERBB receptor family expression at mRNA level in MCF-12A normal breast epithelial cells and in five breast carcinoma cells. B. Western blots showing the expression level of ERBB family receptors. HCC1954 cells express high levels of ERBB1 and ERBB2 receptors, but low level of ERBB3 and no ERBB4. β-actin was used as loading control. C. WST-1 cell viability assay to assess the resistance of breast carcinoma cells to trastuzumab (100 nM). Compared to SK-BR-3 cells, with high level of ERBB2 receptors, HCC1954 cells are resistant to trastuzumab (100 nM) over 4 days. D. Verification of resistance of HCC1954 cells to trastuzumab compared to sensitive SK-BR-3 cells in 3-dimensional cell culture. Photos were taken after eight days of trastuzumab treatment.

We first identified a suitable *de novo *trastuzumab resistant cell system as prerequisite for studying the ERBB-receptor regulated network. This cell system should have high ERBB2 expression but be resistant to trastuzumab treatment. To this end, we first analyzed several breast carcinoma cell lines and the normal epithelial MCF-12A cell line for their expression of ERBB family receptors at mRNA and protein levels, respectively (Figure 1A and 1B). HCC1954 cells, like SK-BR-3 and BT474 cells, express high levels of EGFR (ERBB1) and ERBB2 receptors, but have low levels of ERBB3. ERBB4 receptor expression was not detected in the HCC1954 cell line.

Next, we examined the response to trastuzumab treatment of breast carcinoma cells with high ERBB2 expression (HCC1954 and SK-BR-3) as compared to cells with low ERBB2 level (MCF-7) in a viability assay (Figure 1C). Cells were treated with or without trastuzumab and cell viability was assayed over time to observe the effect of the drug. We further tested different concentrations of trastuzumab to rule out the possibility that the resistance of cell system could have been due to insufficient amounts of trastuzumab being present in the assay (Additional file 1, Figure 1A). While SK-BR-3 cells responded to trastuzumab starting from day two, HCC1954 cells were resistant, as they did not show any response to the drug over four days.

Lastly, we verified the resistance of HCC1954 cells to trastuzumab in 3-D cell culture (Figure 1D). After eight days of treatment, HCC1954 cells were still growing in a large cluster-like structure that was similar to untreated HCC1954 cells, whereas SK-BR-3 cells were sensitive to trastuzumab also in 3-D culture. We could exclude that the resistance phenotype of HCC1954 cells was due to a higher or lower ERBB2 expression level compared to sensitive SK-BR-3 cells (Additional file 1, Figure 1B). Furthermore, to rule out the potential impact of possible mutations in the ERBB2 protein on resistance phenotype of the HCC1954 cells, the *ERBB2 *gene sequence was verified by sequencing and no mutation was found. Hence, HCC1954 cells were chosen as a *de novo *trastuzumab resistant model cell system in this study.

### Determination of experimental output

**Figure 2:**
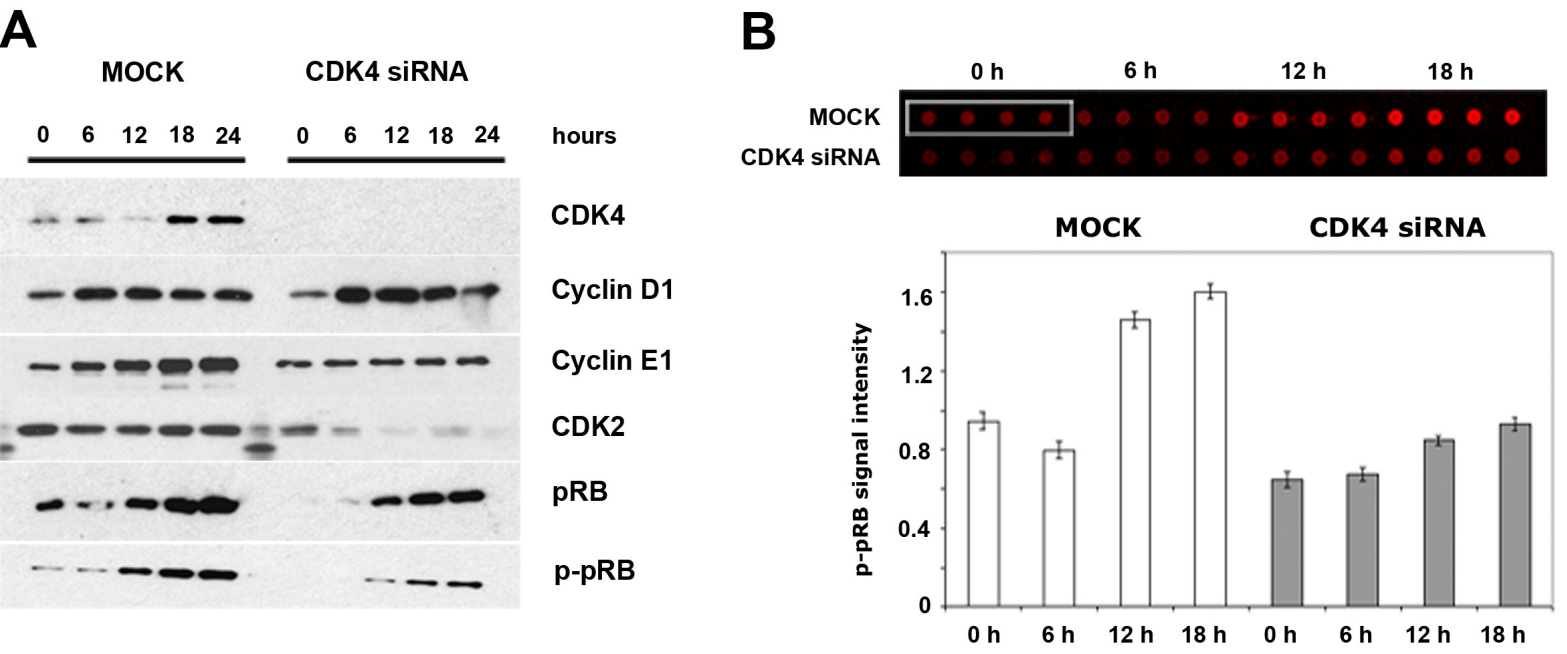
**Proof of principle experiments for the determination of G1/S transition point in trastuzumab resistant HCC1954 cells**. A. Western blots showing the expression and activation of key G1/S proteins. Cells were treated either only with Lipofectamine (MOCK) or with 20 nM CDK4 siRNA for 24 hours. Then, cells were synchronized for 24 hours and subsequently stimulated with 25 ng/ml EGF for 6, 12, 18 or 24 hours. Cell lysates were applied to immunoblotting. B. Reverse Phase Protein Array (RPPA) showing the phosphorylation state of pRB protein (Ser 807/811). The same lysates (from A) were applied to RPPA. The upper panel shows the read-out of antibody signal at near infra-red range for phospho-pRB antibody with four replicates. The lower panel shows the graphical representation of phospho-pRB antibody signal intensity for two different conditions and for four time points. Signals were normalized to 0 hour MOCK sample.

Next, we characterized HCC1954 cells with regard to G1/S progression by measuring the levels of pRB phosphorylation, and of cell cycle proteins by comparing MOCK (only lipofectamine transfection reagent) and CDK4 siRNA transfected cells. After synchronization, we stimulated the cells with EGF. Starting from "0 hour (no EGF)", cells were lysed at different time points and proteins of interest were detected with specific antibodies (Figure 2A). CDK4 knockdown was efficient, as no residual protein was visible on the blot. Due to Dif-3 treatment, which degrades Cyclin D1 at both mRNA and protein levels [26], the level of Cyclin D1 was low at 0 h while it increased upon continuous EGF stimulation. After 6 h of EGF stimulation, Cyclin D1 expression remained constant until 24 hours in both MOCK-treated cells and after CDK4 knockdown. For the MOCK control, a gradual increase in the Cyclin E1 level was observed, starting from EGF stimulation (0 hour) to 18 hours. In contrast, Cyclin E1 expression did not change from 0 h to 18 h after CDK4 knockdown. Surprisingly, we observed a reduction also of CDK2 in the CDK4-siRNA treated cells, starting at 6 h. This might be due to the partnering of CDK2 with Cyclin E1, whose level did not increase in case of CDK4 knockdown.

We found the phosphorylation of pRB (Ser 807/811), our marker for G1/S transition, to be delayed and the pRB expression level to be decreased after CDK4 knockdown, as compared to the MOCK control. Hence, we next quantified the phosphorylation level of pRB (Figure 2B) in the same lysates using reverse phase protein arrays (RPPA). The phosphorylation level of pRB was found to be low in the growth-arrested cells and induction of pRB phosphorylation from 6 to 12 hours did not occur abruptly for CDK4 knockdown compared to MOCK. These data demonstrate that phosphorylation of pRB at the transition point can be quantified by RPPA as an output of EGF stimulation.

### Literature-based Boolean network of G1/S transition

**Figure 3:**
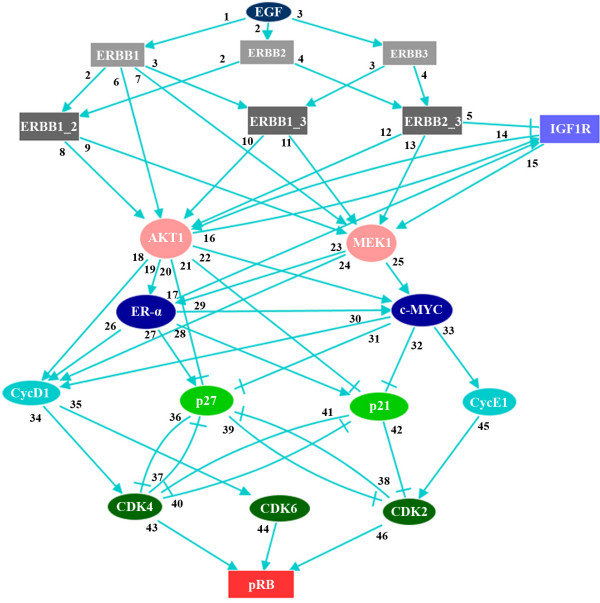
**ERBB receptor regulated G1/S transition network derived from published data**. ERBB receptors are functional (i.e. able to transmit signal to downstream proteins) only when they form heterodimers, except for ERBB1, which is also functional as a homodimer. The number associated with each arrow indicates the reference from which the corresponding interaction was extracted (a list of these references is provided in the Additional file 1, Table 1). Normal arrows denote positive regulations, whereas blunt arrows denote negative regulations. These interactions correspond to transcriptional regulations, post-transcriptional modifications, or physical interaction. EGF constitutes the input and pRB protein represents the output of the network.

The initial network of G1/S transition was built by extracting information from the literature about interactions between proteins involved in receptor tyrosine kinase-regulated cell cycle progression (Additional file 1, Table 1). The resulting network encompasses 18 proteins, including EGF as stimulus, homo- and heterodimers of ERBB family members, tyrosine kinase receptor IGF-1R, key transcription factors (ER-α and c-MYC), key signaling intermediates (AKT1 and MEK1), and G1/S transition cyclins, CDKs and CDK inhibitors (Figure 3). Upon activation, members of the ERBB family of tyrosine kinases form homo- and heterodimers. For HCC1954 cells, six different such dimers are possible as ERBB4 is not expressed in this cell line. The dimers ERBB1/ERBB2, ERBB1/ERBB3 and ERBB2/ERBB3 were represented as specific nodes in the network. Homodimers were implicitly represented by the corresponding protein nodes. Since no ligand is known for ERBB2 homodimers [27] and as ERBB3 homodimers have a defective tyrosine kinase domain [28], the corresponding nodes are unable to activate the ERBB targets AKT1 and MEK1. The effects of the combinations of interactions on the activity of each protein was defined in terms of logical rules using the Boolean operators *AND*, *OR*, and *NOT*. Table 1 lists these Boolean rules for the network components, and data supporting the rules is provided in the Additional file 1, Table 1. We then utilized the modeling and simulation software GINsim [23] to implement these rules into a computational model. Figure 3 shows the resulting logical regulatory graph for the ERBB receptor-regulated G1/S transition protein network. Normal arrows denote positive regulations, which are either through phosphorylation, transcriptional activation, or physical interaction (e.g,. complex formation). Blunt-ended arrows denote negative regulations. The numbers associated with each edge refers to the respective publications providing experimental data in support of the corresponding regulatory interaction (Additional file 1, Table 1).

**Table 1:** Boolean rules for the activation of each component of the network presented in Figure 3.

**Target**	**Logical rules for the activation of target**
ERBB1	EGF
ERBB2	EGF
ERBB3	EGF
ERBB1_2	ERBB1 **Λ **ERBB2
ERBB1_3	ERBB1 **Λ **ERBB3
ERBB2_3	ERBB2 **Λ **ERBB3
IGF1R	(ER-α **V **AKT1) **Λ !**ERBB2_3
ER-α	AKT1 **V **MEK1
c-MYC	AKT1 **V **MEK1 **V **ER-α
AKT1	ERBB1 **V **ERBB1_2 **V **ERBB1_3 **V **ERBB2_3 **V **IGF1R
MEK1	ERBB1 **V **ERBB1_2 **V **ERBB1_3 **V **ERBB2_3 **V **IGF1R
CDK2	Cyclin E1 **Λ !**p21 **Λ !**p27
CDK4	Cyclin D1 **Λ !**p21 **Λ !**p27
CDK6	Cyclin D1
Cyclin D1	AKT1 **V **MEK1 **V **ER-α **V **c-MYC
Cyclin D1*	ER-α **Λ **c-MYC **Λ **(AKT1 **V **MEK1)
Cyclin E1	c-MYC
p21	ER-α **Λ !**AKT1 **Λ !c**-MYC **Λ !**CDK4
p27	ER-α **Λ !**CDK4 **Λ !**CDK2 **Λ !**AKT1 **Λ !c**-MYC
pRB	(CDK4 **Λ **CDK6)**V **(CDK4 **Λ **CDK6 **Λ **CDK2)

These rules were derived from the references listed in the Additional file 1, Table 1. The refined rules for Cyclin D1 are shown with "*". Symbols "Λ": AND, "V": OR and "!": NOT

### Simulation of loss-of-functions

For loss-of-function simulations, we performed *in silico *knockdowns of the network proteins by fixing the level of the perturbed element to "0", meaning that the corresponding protein was always "inactive" (Additional file 1, Figure 2). Each simulation was performed for specific initial protein states, matching the biological and experimental conditions (for several proteins, we considered both possible values). For example, the initial states of p21 and p27, both of which being CDK inhibitors, were set to "1" because, in G0/G1 arrested cells, the expression levels of these inhibitors are high and their levels decrease (due to their degradation in proteasomes) once cells progress through S phase [29]. To represent continuous EGF simulation, the initial values of ERBB nodes were set to "1". Since the cells had been synchronized with Dif-3, which degrades Cyclin D1, the initial level of Cyclin D1 was set to "0". Using the resulting initial states, we computed all possible state transitions and iterated until we finally obtained a unique "stable state" in which the level of each protein was fixed (details about knockdown simulations can be found in Materials and Methods section). Table 2 summarizes the outcomes of the simulations. Out of 17 loss-of-function simulations, a significant decrease of pRB phosphorylation (pRB is predominantly in its hyphophosphorylated form and cells do not progress through G1/S transition: pRB = 0) was predicted for CDK4, Cyclin D1, and CDK6 loss of functions. For the ERBB1_2 and ERBB1_3 knockdowns, we obtained two possible stable states characterized by pRB = 0 and 1 that should be resolved with experimental results. The loss-of-function simulations for all other network proteins resulted in the preservation of pRB phosphorylation (pRB = 1), thus tentatively enabling G1/S transition.

**Table 2:** Comparison of simulation results with experimental data (p-pRB protein data and 7-AAD DNA data).

	**Simulated**	**p-pRB data**	**7-AAD data**	**Improved rules**
MOCK	**1**	**1**	**1**	**1**
AKT1	**1**	**1**	**1**	**1**
MEK1	**1**	**1**	**1**	**1**
CDK2	**1**	**1**	**1**	**1**
CDK4	**0**	**0**	**0**	**0**
p21	**1**	**1**	**1**	**1**
p27	**1**	**1**	**1**	**1**
Cyclin D1	**0**	**0**	**0**	**0**
ERBB2_3	**1**	**1**	**1**	**1**
ERBB1_3	0/1	**1**	**1**	0/1
ERBB1	1	0	1	1
ERBB1_2	0/1	0	1	0/1
IGF1R	1	0	1	1
CDK6	0	0	1	0
ER-α	*1*	**0**	**0**	**0**
c-MYC	*1*	**0**	**0**	**0**
Cyclin E1	*1*	**0**	**0**	*1*

The first column ("Simulated") corresponds to the results of knockdown simulations for the initial logical model, while the last column ("Improved rules") displays the results obtained with the revised model (improved logical rules for Cyclin D1 as shown in Table 1). In the "Simulated", "p-pRB data" and "Improved rules" columns, the value "1" denotes the phosphorylation of pRB (G1/S transition) and "0" denotes the lack of phosphorylation (no G1/S transition). In the "7-AAD data" column, the value "1" denotes a low ratio of G1/S (G1/S transition) and "0" denotes a high G1/S ratio (no G1/S transition). Bold numbers emphasize the knockdowns for which simulations and both experiments agree. Normal numbers correspond to the knockdowns for which experiments led to different values, one of them being met by our simulation results. In the case of ERBB1_3 knockdown, one of the two existing stable states meets the concordant experimental result. The final state depends on the choice of the initial conditions, but these are difficult to fully specify (a similar situation occurs for the ERBB1_2 knockdown). Finally, Italic numbers denote the knockdowns for which simulation data differ from the results of both experiments.

Furthermore, we have also simulated the loss-of-function of multiple proteins (all double and many triple knockdowns) (Additional file 2, Table 1). We have observed that if one protein knockdown gives pRB = 0, the combination of any other protein knockdown with this one also gives pRB = 0 (e.g. ER-α knockdown gives pRB = 0 and ER-α+AKT1 knockdown also gives pRB = 0) and we have verified this experimentally as well (Additional file 2, Table 2). We have also simulated the knockdown of all three receptors at the same time (ERBB1_2_3) and it resulted in 2 stables states with pRB = 1 or 0 (Additional file 2, Table 1).

### Assessment of siRNA knockdown efficiency and specificity for experimental testing of simulations

**Figure 4:**
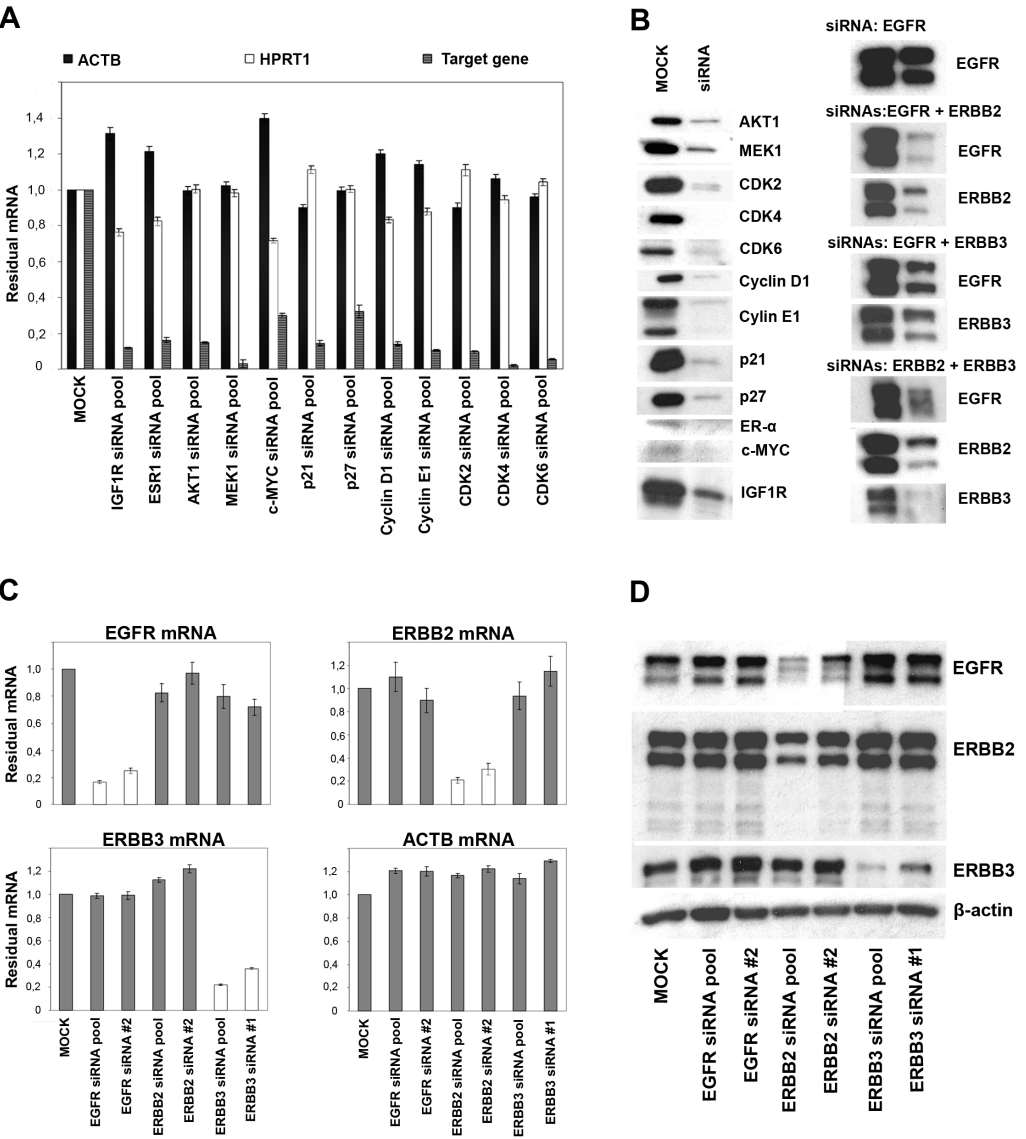
**Determination of knockdown efficiency and specificity of siRNAs in the HCC1954 cell system**. A. qRT-PCR results showing the knockdown efficiency of 20 nM pools of four siRNAs for each gene in the network (50 nM siRNA for *ESR1*). *ACTB *and *HPRT1 *were used as house-keeping controls. MOCK stands for the samples which were treated only with Lipofectamine transfection reagent. B. Western blots for the determination of knockdowns for the network elements at protein level. Since dimers of ERBB family members are accepted as functional units, we used combinatorial RNAi (knockdown of two genes simultaneously) to produce knockdowns of such dimers. C. qRT-PCR results showing the knockdown efficiency and effect of one member of ERBB receptor family siRNA on the other two members of the family. Both pools of four individual siRNAs per gene and only one individual siRNA per gene were used. The concentration of siRNAs was 20 nM. *ACTB *was used as house-keeping control. D. Western blots showing the knockdown efficiency and cross reactivity of siRNAs at protein level. β-actin was taken as loading control.

In order to validate the simulations having been performed for various possible loss of functions, we utilized RNAi to experimentally induce knockdown of the corresponding proteins. First, we validated the siRNAs according to their knockdown efficiency at both mRNA and protein levels by qRT-PCR and Western blotting, respectively (Figure 4A and 4B). We obtained at least 70% knockdown at mRNA level for all the network proteins (Figure 4A) and for most a similar knockdown also at the protein level (Figure 4B), both in single and combinatorial RNAi settings [24]. Because of the high level of sequence conservation among ERBB family receptors [27], it was imperative to test for a potential cross-reactivity of ERBB receptor siRNAs (Figure 4C and 4D). To this end, we compared the effects of the pools of four siRNAs for every gene with those of individual siRNAs. Neither pools nor individual siRNAs were found to have cross-reactivity (Figure 4C and 4D).

In the combinatorial RNAi settings, the levels of the ERBB proteins in the EGFR/ERBB2, EGFR/ERBB3, and ERBB2/ERBB3 heterodimers were efficiently downregulated (Figure 4B). While the EGFR level was drastically reduced when we applied double knockdown of EGFR/ERBB2, EGFR/ERBB3, and ERBB2/ERBB3 (Figure 4B), the EGFR protein was stable in the single knockdown with EGFR siRNA although this treatment resulted in more than 80% knockdown at the mRNA level (Figure 4A). However, knockdown of the ERBB2 receptor resulted in a substantial decrease of EGFR at the protein level. With respect to the qRT-PCR results, we can exclude that this effect could be due to a cross-reaction of the ERBB2 siRNA (Figure 4C). Therefore, we hypothesize an indispensable partnering of ERBB2 and EGFR in ERBB2 overexpressing cells (Figure 4D), and assume that the EGFR receptor protein is efficiently stabilized that way. This hypothesis was supported by similar observations made in ERBB2 overexpressing SK-BR-3 cells, but not in MDA-MB-231 having low ERBB2 expression (Additional file 1, Figure 3).

### Experimental validation of loss-of-function simulations

**Figure 5:**
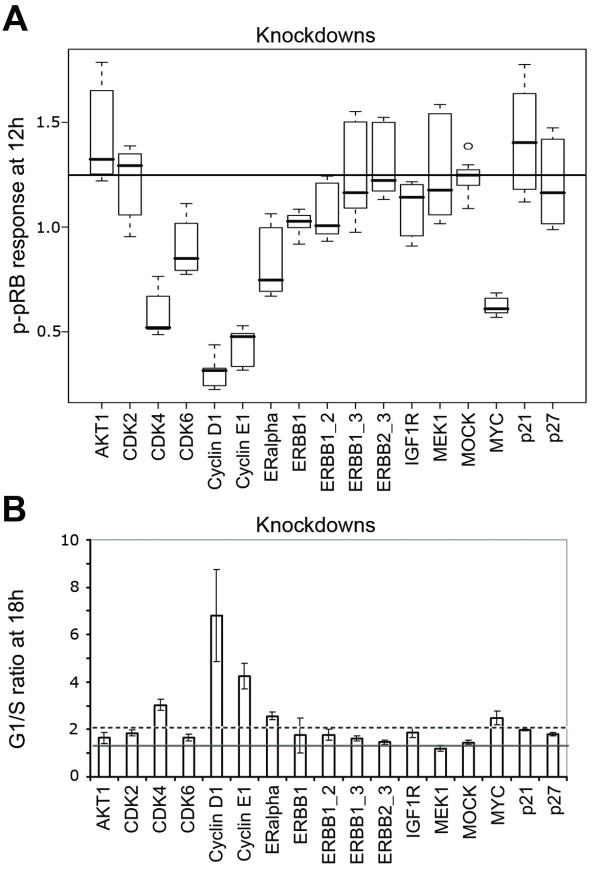
**Analysis of the effects of knockdowns on G1/S transition in trastuzumab resistant HCC1954 cells**. A. Phosphorylation state of pRB (output of the network) after 12 hour EGF stimulation (input of the network). Box plots show the quantitative phosphorylation of pRB protein for the knockdown of each network protein compared to MOCK (only transfection reagent, no siRNA). B. G1/S ratio after 18 hours of EGF stimulation for corresponding knockdowns compared to MOCK (solid line) and p21 knockdown (dotted line) using 7-AAD DNA staining.

Next, we designed a series of *in vitro *experiments using the validated conditions described above to assess the results from loss of function simulations. Lysates of three biological replicates were analyzed with RPPA using four technical replicates of each. The signal intensity of phosphorylated pRB was measured in the near-infrared range (NIR) for each knockdown at two time points (0 h and 12 h). As a negative control, we utilized MOCK samples which had not been stimulated with EGF. Results were compared to MOCK samples (reference sample), which had been stimulated with EGF, and the significance of the impact on pRB phosphorylation was tested using the ANOVA method. Box plots of the knockdown effect on pRB phosphorylation are shown in Figure 5A. We classified effects as "1" in cases where the knockdown of a specific protein had resulted in a phosphorylation profile similar to the MOCK profile at 12 h, and no significant change of the pRB phosphorylation state had been observed. Conversely, if the knockdown resulted in a significantly lower pRB phosphorylation level compared to MOCK (FDR < 1%), the effect was classified as "0", meaning that a significant change of the pRB phosphorylation state was observed. The results demonstrated that knockdowns of CDK4, CDK6, Cyclin D1, Cyclin E1, ER-α, c-MYC, ERBB1, ERBB1_2, and IGF-1R indeed resulted in a significant hypophosphorylation of pRB.

Simulation of loss-of-function of all three receptors (ERB1_2_3) had resulted in two stable states for pRB: "1" and "0". Experimentally, we have shown that knockdown of all three receptors resulted in pRB = 1 suggesting that combinatorial targeting of ERBB receptors may not be beneficial to overcome resistance in *de novo *trastuzumab resistant cells (Additional file 2, Table 2).

To confirm the effect of knockdowns on G1/S transition at the DNA level, we measured incorporation of 7-AAD into the DNA of single cells by flow cytometry 18 hours after EGF stimulation (Figure 5B). The fractions of cells in G1- and in S phases were taken to calculate the G1/S ratio for each knockdown. We regarded gene knockdowns having similar effects as MOCK or p21 to be positive for G1/S transition ("value 1"), while G1/S ratios higher than MOCK or p21 were considered negative ("value 0"). For the knockdowns of CDK4, Cyclin D1, Cyclin E1, ER-α and c-MYC, we thus observed no G1/S transition (Table 2).

Table 2 summarizes the simulation results for the final state of the pRB protein and the respective experimental data. Indeed, 12 out of 17 knockdown simulations were consistent with experimentally measured pRB activity levels (compare columns 1 and 2 of Table 2). The correlation is even slightly better with 7-AAD data, as the results of 13 out of 17 simulations were consistent with those of the p-pRB data (compare columns 1 and 3 of Table 2). However, the simulations of ER-α, c-MYC and Cyclin E1 knockdowns gave results that are inconsistent with both types of the experimental data, pointing out the limits of our current model, which we will address in the next section. Altogether, our results suggest that Cyclin D1, CDK4, Cyclin E1, ER-α and c-MYC, but neither the combinatorial targeting of ERBB family receptors nor of key components of the MAPK (MEK1) and the survival pathways (AKT1) should be considered as potential targets for further testing in our *de novo *trastuzumab resistant model cell system.

### Refinement of logical rules and network reconstruction based on quantitative protein data

To find out whether the observed discrepancies between our network model and the experimental data were due to the incorporation of incorrect logical rules, missing interactions, or even missing components in our literature-based network, we next refined the logical rules and performed a network reconstruction that was based on quantitative protein data.

Extracting information about combinatorial regulatory effects of different proteins affecting a given component is much more difficult than extracting information about individual interactions from the literature. We have thus systematically evaluated modifications of the logical rule with respect to model prediction capacity. In particular, the discrepancy between the simulation results and experimental counterparts for c-MYC and ER-α knockdowns could be solved by changing the logical rules associated with the Cyclin D1 node. We tested several combinations for the logical rules on ER-α, c-MYC and Cyclin D1. A minor modification of the model enabled us to recover the correct behaviour for the two loss-of-functions: ER-α and c-MYC (with a stable state having pRB = 0), while conserving all the behaviour for all other proteins. The original rules assumed that the presence of one activator is sufficient (the OR connecting all 4 variables in Table 2). This is the loosest rule that can be defined for a node that is activated by several regulators. The modified rule (Cyclin D1 = 1 when ER-α *AND* c-MYC *AND* (AKT1 *OR *MEK1) is more restrictive as this states that that ER-α AND c-MYC together with AKT1 OR MEK1, are required to activate Cyclin D1. The biological implication of this change is that ER-α, c-MYC and (MEK1 or AKT1) proteins should act together to make the cells pass through S-phase and proliferate. In addition, although both transcription factors are necessary at the same time, the function of the one of the signaling molecule, AKT1 or MEK1, can be compensated in the cell, but not of the two at the same time. So, our results may propose a more comprehensive logic for the regulation of Cyclin D1 in our model cell system. These results may hint that control of Cyclin D1 is a sequential event (AKT1 or MEK1 → ER-α → c-MYC) and can exclude the alternative edges from ER-α, MEK1 or AKT1 to Cyclin D1. We are thus left with just one knockdown simulation (Cyclin E1) disagreeing with the experimental data.

**Figure 6:**
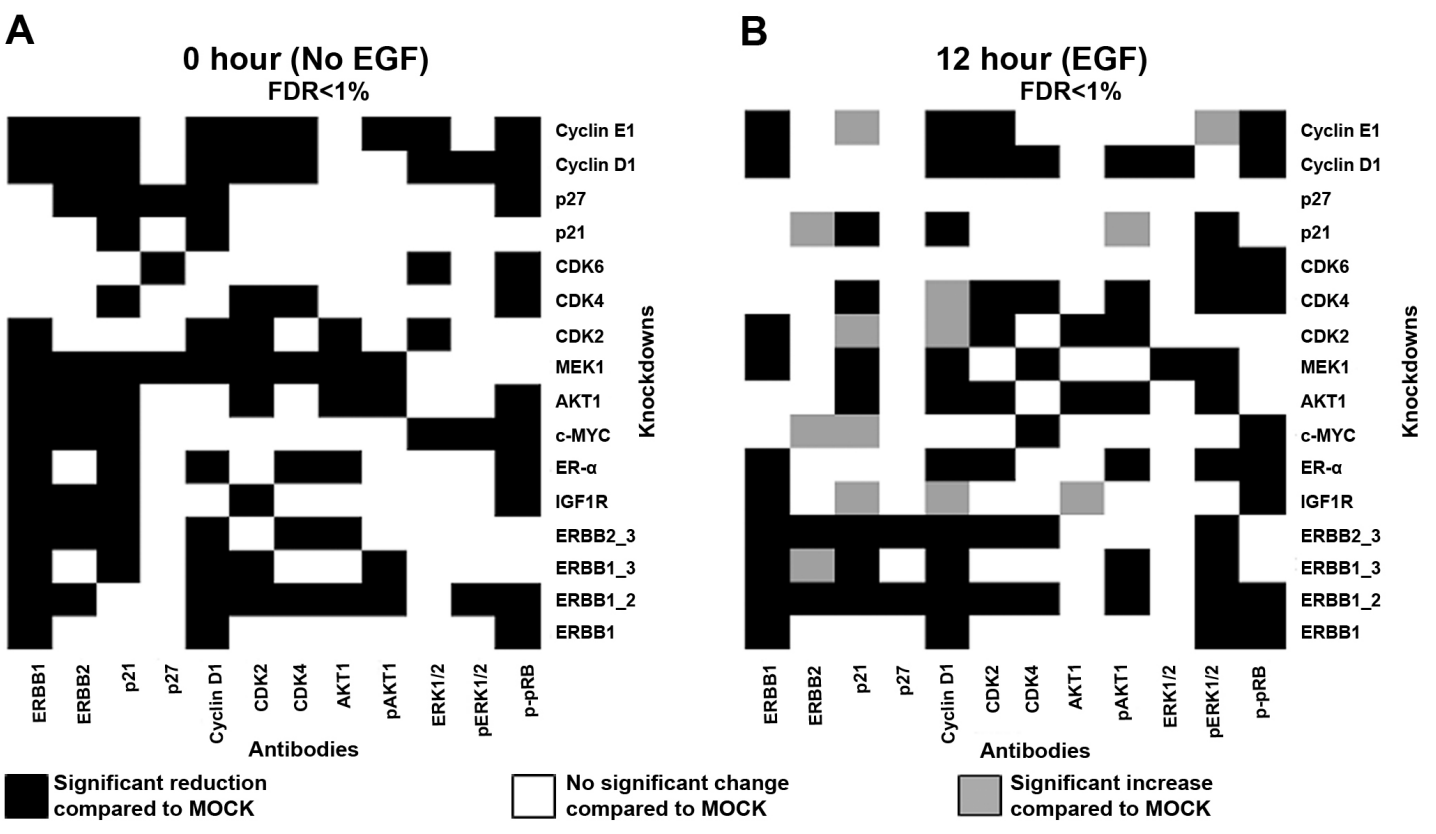
**Response of network proteins for corresponding knockdowns in trastuzumab resistant HCC1954 breast carcinoma cells (A) before and (B) after EGF stimulation**. Heatmaps were drawn for a false discovery rate (FDR) of less than 1%.

**Figure 7:**
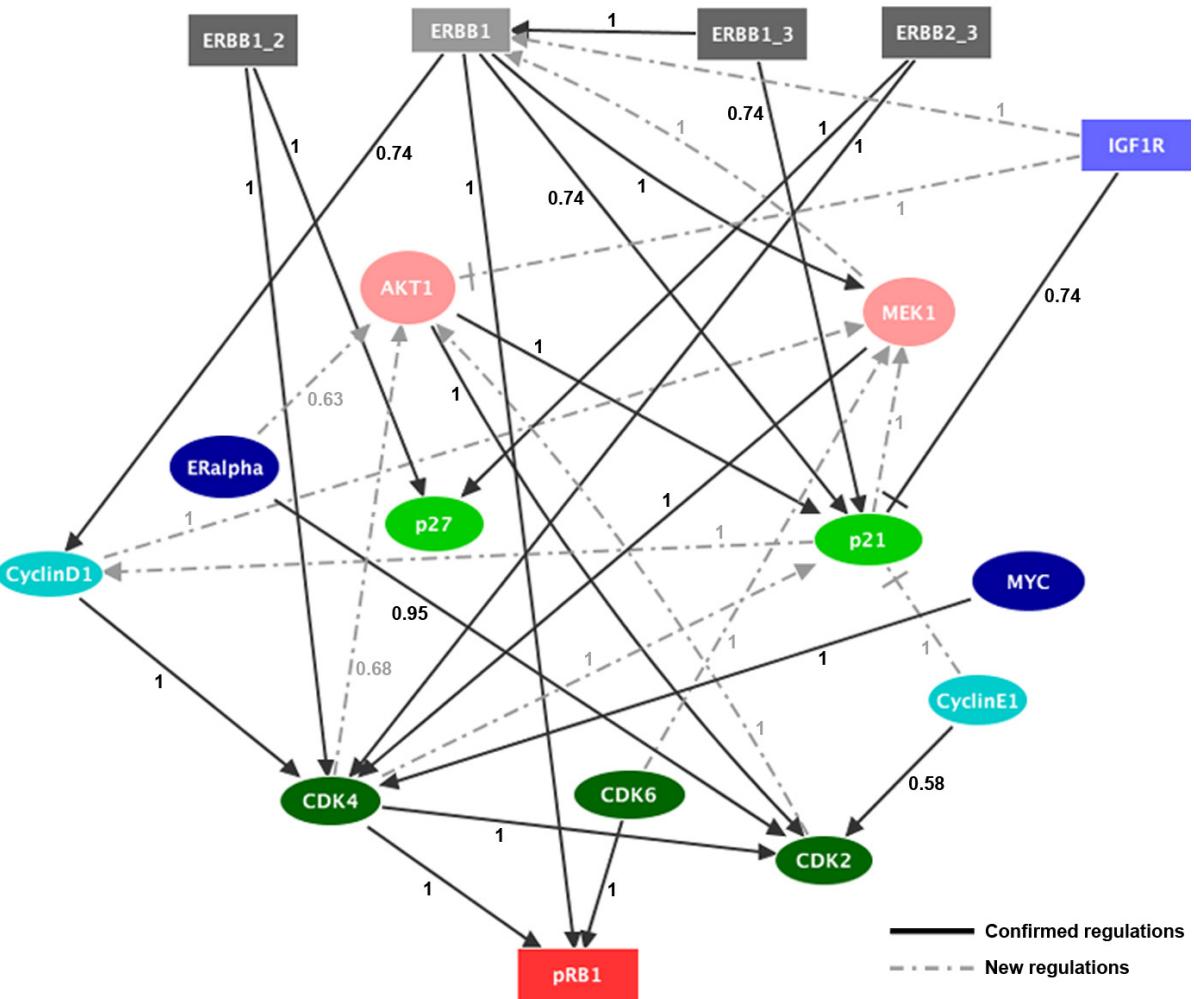
**Tentative regulatory interactions inferred from protein expression and activation data for trastuzumab resistant HCC1954 cells**. The heatmaps in Figure 6B were used to infer the interactions of proteins in the HCC1954 cell system with a probability higher than 50%. The numbers next to each arrow indicate the probability of each interaction. Solid black arrows denote the interactions (both direct and indirect) supported by published data, whereas dotted grey arrows denote novel interactions (compare with Figure 3). Normal arrows denote positive regulations, whereas blunt arrows denote negative regulations.

In the next step, we wanted to test if the discrepancy observed in the case of Cyclin E1 knockdown could be attributed to some missing interactions among the regulatory components considered in our logical model. In order to address this discrepancy and also to examine cell line specific regulations, we further quantified the activation and expression levels of most of the network elements for individual and combinatorial network protein knockdowns using reverse phase protein arrays. In total, we quantified the changes in expression of nine network proteins, as well as the phosphorylation levels of ERK1/2 and AKT1. Some proteins could not be included in these measurements because of the lack of antibodies suitable for RPPA. As for the pRB experiments, we examined the effect of EGF stimulation on the other network proteins for each knockdown, compared to MOCK. The heatmaps in Figure 6 show the significant changes, at the expression or activation level of the proteins before EGF stimulation (Figure 6A) and 12 h after EGF stimulation (Figure 6B). Expression and phosphorylation levels either confirmed known interactions or inferred novel ones (Figure 6B). The resulting interactions define the network presented in Figure 7. A jackknife procedure (see Methods section) was used to eliminate putative indirect edges, which could be explained by a path along other edges, and only edges having a jackknife probability greater than 50% were kept (Figure 7). In the graph, solid black arrows indicate inferred direct or indirect interactions which are also supported by published data, whereas the dotted grey arrows denote novel regulations having been identified for the HCC1954 reference cellular system. As a result, we inferred most of the interactions considered in our literature-based network from the experimental data for trastuzumab resistant HCC1954 cells, although some of them have opposite directions. One should also take into account that drawing edge directions from biological literature is usually a daunting task. Indeed, edge directions, when indicated, are often not well defined or even erroneous. Additionally, there can be cell line specific differences or thus far unknown feedback mechanisms, for example a feedback from Cyclin D1 to MEK1 or from ER-α to AKT1. It is, therefore, not really unlikely to see edges that link in the direction opposite to the expected. Hence, although this approach demonstrates the feasibility of network reverse engineering at protein level using robust and quantitative protein array data, the resulting network was no help to solve the discrepancy observed for Cyclin E1 knockdown, thereby leaving a gap in our knowledge in the regulation or regulatory effects of this component. To sum up, our network inference approach provided us with the knowledge that the most regulations which were obtained from the literature were also present in our trastuzumab cell system and novel regulations might have potential role in the observed phenotype of the cells.

### Combinatorial targeting of c-MYC or EGFR in combination with ERBB2 using small chemical inhibitors in sensitive and resistant cells

**Figure 8:**
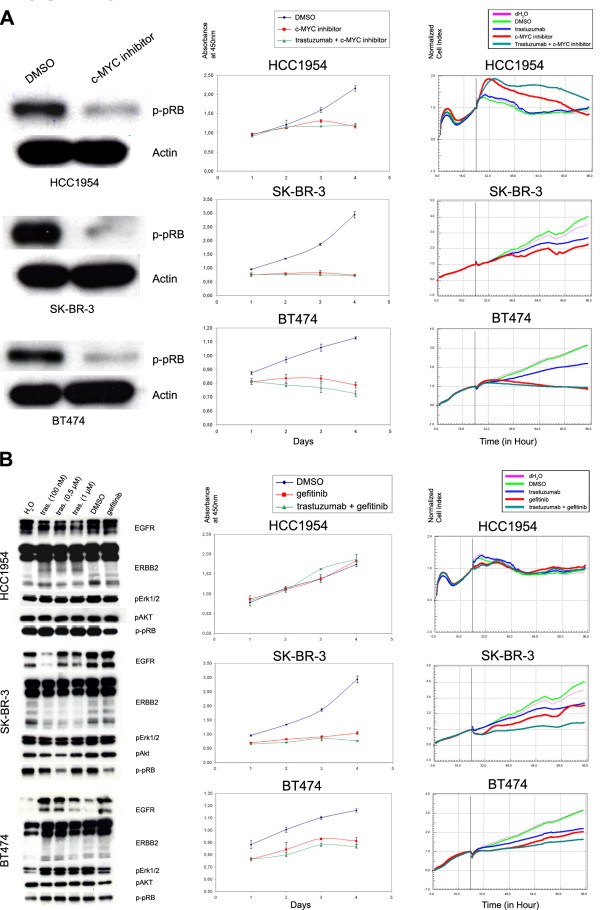
**Effects of c-MYC inhibitor 10058-F4 and EGFR inhibitor gefitinib alone or in combination with trastuzumab on the viability of resistant (HCC1954) and sensitive (SK-BR-3 and BT474) cells**. A. *Left panel: *Western blot data showing the phosphorylation state of pRB protein after treatment with c-MYC inhibitor (10058-F4, 80 μM) for 24 hours. *Middle panel: *WST-1 viability assay over 4 days to determine the response of cells to c-MYC inhibitor alone and in combination with trastuzumab. DMSO is used as vehicle control. *Right panel: *Impedance measurement for real-time determination of cell growth before (24 hours) and after (72 hours) treatment of cells with c-MYC inhibitor, trastuzumab, and the combination of both drugs. The impedance measurements were normalized to the time point where we have added the drugs. The vertical line demonstrates the time point of normalization. B. *Left panel: *Western blot data showing the effect of different concentrations of trastuzumab and gefitinib (1 μM) on the expression levels of EGFR and ERBB2 and on the phosphorylation states of ERK1/2, AKT and pRB proteins. H_2_O is vehicle control for trastuzumab and DMSO is for gefitinib. *Middle panel: *WST-1 assay to determine the response of cells to gefitinib and gefitinib + trastuzumab. *Right panel: *The impedance measurement for real-time measurement of cell growth before (24 hours) and after (72 hours) treatment of cells with gefitinib, trastuzumab and combination of both drugs. Normalization of cell index is as in A.

In order to verify the RNAi results and to validate the potential targets in our *de novo *resistant cell system, we applied small chemical inhibitors against c-MYC (10058-F4) and EGFR (gefitinib), alone and in combination with trastuzumab, and examined the growth of trastuzumab resistant HCC1954 cells compared to sensitive SK-BR-3 and BT474 cells. Administration of the c-MYC inhibitor alone resulted in reduced pRB phosphorylation in all three cells lines (Figure 8A, left panel), and its application alone or in combination with trastuzumab also reduced the growth of these cell lines (Figure 8B, middle panel). The results were verified using real-time impedance measurements over four days (Figure 8C, right panel), providing a time-lapse profile of the growth rates. The resulting data demonstrate that the reduced growth rates of cells treated with the c-MYC inhibitor was independent from trastuzumab resistance and thus support the RNAi results shown in Figure 5A and 5B.

Because combinatorial targeting of ERBB receptors is already in clinical use (e.g. lapatinib), we next targeted ERBB1 and ERBB2 receptors in single and combinatorial settings and compared the outcome in the *de novo *resistant HCC1954 cell line with the trastuzumab sensitive cell lines. First, we examined the downstream effectors of ERBB receptors (ERK1/2 and AKT1) and of pRB after treatment with trastuzumab or gefitinib (targets ERBB1) (Figure 8B, left panel). In HCC1954 cells, no reduction in the expression levels of EGFR/ERBB2 or phosphorylation levels of their downstream signal mediators was observed for both treatments. However, reduced pRB phosphorylation was observed in gefitinib-treated HCC1954 cells. Both, the WST-1 viability assay and real-time impedance measurements demonstrated that trastuzumab resistant HCC1954 cells were also resistant to gefitinib treatment alone or in combination with trastuzumab. (Figure 8B, middle and right panel).

In SK-BR-3 cells, AKT1 and pRB phosphorylation levels were lower after trastuzumab treatment, and pRB phosphorylation was reduced after incubation with gefitinib. In BT474 cells, a strong reduction in AKT and ERK1/2 phosphorylation was measured after gefitinib treatment, while no such an effect was evident for trastuzumab treatment (Figure 8B, left panel). Real-time impedance measurement showed for the SK-BR-3 cells that combinatorial targeting of EGFR and ERBB2 had a strong additive effect to reduce cell proliferation (Figure 8B, right panel). This additive effect was also visible for BT474 cells although it was not as strong as in SK-BR-3 cells. These data support our RNAi results, suggesting that the combinatorial targeting of the EGFR and ERBB2 with gefitinib and trastuzumab, respectively, might not be effective to sensitize the cells to trastuzumab treatment in *de novo *trastuzumab resistance. However, these drugs in combination might lead to an improved outcome in sensitive cells, and potentially also in tumors, as compared to applying them individually.

## Discussion

In the present study, we have applied a systems biology approach to identify alternative targets in *de novo *trastuzumab resistant breast cancer. While several studies have dealt with mechanisms leading to acquired trastuzumab resistance, there has been no comprehensive study that searched for targets alternative to ERBB2 in *de novo *trastuzumab resistance. Since the aim of cancer therapy is to reduce the growth rate of cancer cells, and trastuzumab resistant breast cancer cells escape cell cycle arrest during treatment, we focused on a protein network that connects ERBB signaling to G1/S phase transition, in order to determine new potential targets for perturbation. In contrast to previous studies, which had focused on the involvement of an individual protein in the resistant phenotype of the cells, we aimed to examine the roles of each protein in the context of their interactions at a protein network level.

Several modeling studies about the ERBB receptor-regulated signaling pathways have been published recently [30,31]. These studies considered the activation of key intermediates (ERK1/2 and AKT) upon EGF and HRG stimulation and proposed differential dynamical models for these pathways. Likewise, various models have been proposed for the control of the mammalian cell cycle [18,22,32]. In our study, we combined these two cellular processes into one coherent network to find novel strategies for breast cancer therapy. First, we derived a logical network from published data (Figure 3 and Table 1). Systematic simulations of loss-of-function perturbations were performed, and the final state of pRB phosphorylation (the marker for G1/S transition) was determined in each case. These computational results were then compared with experimental knockdowns, obtained by RNA interference. While each network component was targeted by siRNAs in a single knockdown setting, both constituents of ERBB heterodimers were repressed in a combinatorial RNAi setting (Figure 4). We quantified the effects of these knockdowns on pRB phosphorylation with reverse phase protein arrays (RPPA) (Figure 2 and Figure 5). Knockdowns of c-MYC, ER-α and Cyclin E1 resulted in a very strong reduction in pRB phosphorylation compared to ERBB1, ERBB1/ERBB2, and IGF1R knockdowns (Figure 5A). We then determined the ratio of G1/S to further verify the effect of these knockdowns on G1/S transition (Figure 5B). DNA staining enabled us to differentiate the strong effects of c-MYC, ER-α and Cyclin E1 knockdown as compared to weaker effects of ERBB1, ERBB1/ERBB2, and IGF1R knockdowns (Figure 5B).

Consequently, Cyclin D1 and CDK4 were identified as potential targets from both simulation and experiments. This result was expected, as in response to an external stimulus, Cyclin D1 and CDK4 make a complex that phosphorylates pRB, and which, in turn, enables G1/S transition. Accordingly, we found knockdown of these proteins to result in a significant reduction in the phosphorylation of pRB. In contrast, c-MYC, ER-α and Cyclin E1 were identified by experimental analyses on *de novo *trastuzumab resistant cells, but had not been predicted in the initial network model. After network refinement, c-MYC and ER-α were also predicted as targets from the model (Table 1 and 2). Hence, we demonstrated that this approach enables the reconstruction of phenotype-specific interactions, which are essential to predict therapeutic strategies.

In addition, missing components in the protein network can also be inferred. For example, while Cyclin E1 and CDK2 form a complex, which further phosphorylates the pRB protein, our experimental data show that only loss of Cyclin E1, but not of CDK2, significantly repressed phosphorylation of pRB (Figure 5A). This result suggests that CDK2 could be a dispensable component for the G1/S transition in *de novo *trastuzumab resistant breast cancer, as it has previously been shown for colon cancer cells [33]. This observation raises the question which alternative interaction partners of Cyclin E1 could promote G1/S transition.

In our study, the transcription factors c-MYC and ER-α were identified as potential targets to overcome *de novo *trastuzumab resistance. Park *et al *had previously shown that an amplification of the c-MYC gene is correlated with ERBB2 overexpression in breast cancer [34]. In trastuzumab sensitive cells, ERBB2-targeted antibodies can inhibit c-MYC through inhibition of the MAPK and AKT pathway which, in turn, increases the activity of p27 towards the CDK2-Cyclin E complex [35]. Here, we demonstrated that loss of c-MYC activity results in a reduction of the CDK4 level which then results in reduced pRB phosphorylation (Figure 7). Targeting c-MYC with a specific chemical inhibitor alone or in combination with trastuzumab also resulted in a strong reduction in pRB phosphorylation and cell growth, both in trastuzumab resistant and sensitive cells (Figure 8A). We conclude that targeting c-MYC alone or in combination with trastuzumab could be an interesting candidate for a clinical trial. Cross-talk between ERBB2 signaling and ER-α activation has been previously reported [36], and an increase in the ERBB2 expression level has been reported in tamoxifen resistant cells [37]. In this study, we have shown that ER-α is another possible target in ERBB2 overexpressing and trastuzumab resistant HCC1954 cells. This suggests an interplay between ER-α and ERBB2 receptors in the context of bypassing the effects of drug treatment.

Interestingly, the five novel candidates to be targeted in *de novo *trastuzumab resistant breast cancer have one feature in common: they all either directly (ER-α, c-MYC and CDK4) or indirectly (Cyclin D1 and Cyclin E1) regulate the p27 protein, which plays a key role also in acquired trastuzumab resistance [38]. In addition, our study indicated that combinatorial targeting of either of ERBB1, ERBB2 and ERRB3 may not enhance sensitivity to trastuzumab in *de novo *resistant patients, although ERBB proteins have been previously considered as promising targets. These results let us hypothesize that these cell surface proteins (here: ERBB receptors or IGF1R) are decoupled from intracellular processes (here: G1/S transition) in the *de novo *trastuzumab resistant cell system. Targeting EGFR alone or in combination with ERBB2 further supported this notion. While trastuzumab sensitive cells (SK-BR-3 and BT474) were responding to gefitinib as well as combination of gefitinib and trastuzumab treatment in an additive manner, *de novo *trastuzumab resistant cells (HCC1954) did not respond at all (Figure 8B). This observation suggests that combinatorial targeting of cell surface receptors might be beneficial as it is an uncommon perturbation for cells [39], but it should be taken into consideration that this might be cell system- or patient specific.

According to our results, targeting EGFR with siRNAs alone resulted in an efficient knockdown at the mRNA level; however, no reduction was observed at the protein level (Figure 4). This phenomenon might be explained by the stabilization of EGFR after dimerization with the overexpressed ERBB2 receptor. Complementary to this observation, knocking down ERBB2 resulted in reduced EGFR expression at the protein level, although no reduction of EGFR was observed at the mRNA level (Figure 4). These data demonstrate an explicit dependence of EGFR protein abundance on ERBB2 expression, and should be kept in mind when EGFR is targeted in cancer therapies. This observation is independent of trastuzumab sensitivity, but is highly influenced by the ERBB2 expression level (Figure 1 and Additional file 1, Figure 3).

It should be noted that the logical formalism used in this work clearly caricatures subtle dose effects into all-or-none responses for all the components considered. The resulting logical model should thus be taken as a first step in the formalisation of the regulatory network involved in trastuzumab resistance. However, it is appropriate to translate qualitative information and compare the behaviour of alternative network wirings or logical rules with data sets for unperturbed and perturbed situations. Once the regulatory wiring and the logical rules be reasonably established, it will be possible to take advantage of multi-level logical modeling extensions, or yet to translate our Boolean models into more quantitative formalisms (e.g. ordinary differential equations, or yet hybrid or stochastic Petri nets).

## Conclusion

We constructed a literature-based protein network and combined computational simulations, validation experiments using RNAi, as well as chemical inhibitors, and network inference based on proteomic data, in order to identify novel targets with potential for individual and combinatorial therapies in breast cancer. Our concept to combine experimental and computational biology demonstrated the strengths and limitations of using literature-based models for simulations of therapeutic strategies. Furthermore, this study led us to select c-MYC as a candidate to be tested in *in vitro *and *in vivo *models, regarding future treatments for breast cancer which is *de novo *resistant to trastuzumab. Our results also suggest that combinatorial targeting of key ERBB receptors might have better outcome than individual therapies in trastuzumab sensitive cells, but not in *de novo *trastuzumab resistant cells.

## Methods

### Cell Culture

Five human breast cancer cell lines (HCC1954, SK-BR-3, MDA-MB-231, BT474 and MCF-7) as well as the normal breast epithelial cell line MCF-12A were obtained from ATCC (Manassas, VA). HCC1954 cells (CRL-2338) were cultured in RPMI 1640 Modified Medium (ATCC), SK-BR-3 cells (HTB-30) in McCoy's 5a medium (GIBCO BRL), and MDA-MB-231 cells (HTB-26) in Leibovitz's L-15 medium (Sigma). BT474 cells (HTB-20) were cultured in DMEM medium, MCF-7 cells (HTB-22) in Eagle's Minimial essential medium, and MCF-12A cells (CRL-10782) in a medium containing a 1:2 mixture of Dulbecco's Modified Eagle's Medium and Ham's F12 medium. All media were supplemented with 50 U/mL penicillin, 50 μg/mL streptomycin sulphate, 1% non-essential amino acids and 10% fetal bovine serum (all media and supplements from Gibco BRL). Additionally, 2.2 g/L sodium bicarbonate was supplemented for MDA-MB-231 cells. Media for BT474 cells were supplemented with 10% NCTC medium, 500 μl bovine insulin and 100 μl Oxalic acid, for MCF-7 cells we added 0.01 mg/mL bovine insulin, and such for MCF-12A cells were supplemented with 20 ng/mL EGF, 100 ng/mL cholera toxin, 0.01 mg/mL bovine insulin, and 500 ng/mL hydrocortisone. The cells were incubated at 37°C with 5% CO_2 _and split 2–3 times per week in a 1:3 ratio for no more than 20 passages. All cell lines were validated by genotyping.

### 3-D cell culture

HCC1954 and SK-BR-3 cells were cultured in 8-well chamber slides in order to examine the effect of trastuzumab on proliferation. Geltrex (Invitrogen, Carlsbad, CA) was thawed on ice and 40 μL was pipetted per well and left for 30 min at 37°C to solidify. HCC1954 and SK-BR-3 (5.000 cells/well) cells were seeded in medium supplemented with 1:50 Geltrex and EGF (BD Biosciences), and with or without trastuzumab (100 nM) (Roche, Penzberg, Germany). The cells were incubated at 37°C and 5% CO_2 _for eight days, and medium was changed after 4 days.

### siRNA transfections and EGF stimulations

HCC1954 cells were seeded at a number of 7 × 10^5 ^cells per 10 cm petri dish in antibiotic free medium. Confluency of the cells was 50–60% at the day of transfection. Sequences of siRNAs are given in Additional file 1, Table 2. Twenty nM of siRNA (except *ESR1 *siRNA (50 nM)) (Dharmacon, Lafayette, CO) and 25 μL of Lipofectamine 2000™ (Invitrogen, Carlsbad, CA) were diluted separately in reduced-serum medium OptiMEM (Gibco BRL) and incubated for 5 minutes at RT. The two solutions were then mixed and incubated for 20 minutes at RT. The siRNA-Lipofectamine 2000™ mixture was then added to the cells and the dishes were shaken by gentle rocking. MOCK transfected cells were treated with Lipofectamine 2000, but no siRNA was added. The cells were incubated at 37°C and 5% CO_2 _for 24 hours. After incubation, cells were starved by Dif-3 (30 μM) (Sigma) for 22 hours in medium containing 10% FBS. Cells were further starved in 0% FBS medium for 2 hours. After 24 hours of starvation, cells were stimulated with EGF (25 ng/mL) for 6, 12, 18 and 24 hours.

### Cell lysis and Western blotting

At each time point, medium was removed and cells were washed with ice-cold PBS containing 10 mM NaF and 1 mM Na_4_VO_3_. Lysis of cells was performed on ice by scraping or by cold trypsinization, and shaking on over-head shaker for 15 minutes at 4°C with 70 μl M-PER lysis buffer (Pierce, Rockford, IL) containing protease inhibitor Complete Mini (Roche, Basel), anti-phosphatase PhosSTOP (Roche, Basel), 10 mM NaF and 1 mM Na_4_VO_3_. Protein concentrations were determined with a BCA Protein Assay Reagent Kit (Pierce, Rockford, IL). Proteins were denaturated with 4× Roti Load (Roth, Karlsruhe, Germany) at 95°C for 5 minutes, and 12 μg proteins were loaded in every lane. Protein samples were separated by 8% or 12% SDS PAGE, electroblotted to PVDF membranes (Amersham Biosciences, USA) and exposed to primary antibodies. A list of antibodies is given in Additional file 1, Table 3, together with their dilutions. Horseradish peroxidase conjugated anti-mouse or rabbit antibodies (Amersham Biosciences, USA) were used as secondary antibodies and signals were detected by enhanced chemiluminescence (Amersham Biosciences, USA).

### TaqMan (qRT-PCR)

Total RNA was extracted from the cells by using the Invisorb Spin cell RNA mini kit (Invitek GmbH, Berlin, Germany), and single-stranded cDNA was transcribed with the RevertAid H Minus First Strand cDNA Synthesis kit (Fermentas, St. Leon-Rot, Germany). Ten nanograms of total RNA were used for each reaction. qRT-PCR for target genes and housekeeping genes ACTB and HPRT1 was performed with the ABI Prism 7900HT Sequence Detection System (Applied Biosystems, Weiterstadt, Germany), applying probes of the Universal Probe Library (Roche, Penzberg, Germany). Primers were synthesized by MWG (Ebersberg, Germany). Sequences of primers and the respective UPL probe numbers are given in Additional file 1, Table 4.

### Reverse Phase Protein Arrays (RPPA)

Cell lysates were prepared as for Western Blotting. All lysates were adjusted to a total protein concentration of 3 μg/μl. Cell lysates were mixed 1:2 with 2× Protein Arraying Buffer (Whatman, Brentfort, UK) to yield a final protein concentration of 1.5 μg/μL. The samples were printed with a non-contact piezo spotter, sciFlexxarrayer S5 (Scienion, Berlin, Germany), in four replicate spots per sample and subarray, and two subarrays per slide onto nitrocellulose coated ONCYTE-slides (Grace Bio Labs, Bend, USA). Twenty replicate slides were produced per run. Approximately 2.25 ng total proteins were delivered per spot. As spotting control, the total protein content of all spots was determined for two replicate slides with the FAST Green FCF assay. All antibody signals were normalized according to their total protein content. Slides were blocked over night and target proteins were detected with specific primary antibodies (Additional file 1, Table 3) using a protein array incubation chamber (n1-quadrat, Metecon, Mannheim, Germany). Detection of primary antibodies was carried out with near-infrared (NIR)-dye labeled secondary antibodies and visualized using an Odyssey scanner (LI-COR, Lincoln, USA). Signal intensities were quantified using Odyssey 2.0 software, corrected for spot-specific background signals and normalized for their total protein concentrations.

### 7-AAD staining and analysis

Directly after siRNA transfections, cells were synchronized for 24 hours (see above), and stimulated with EGF for 18 hours. Then, cells were trypsinized, washed once with PBS and centrifuged. Ice cold methanol was added to the cell pellets while vortexing the FACS tube. After incubation of cells at -20°C overnight, methanol was removed and 250 μl of 7-AAD (1:40 dilution) (Calbiochem, Darmstadt, Germany) was added to each tube and incubated for 1.5 hours at 4°C in the dark. The measurement was done by flow cytometry (FACS Calibur, BD Biosciences) using the FL3 channel for 7-AAD staining. Analysis of the 7-AAD results was performed using CellQuest Pro software with the histogram statistics option and a gate on the main cell population.

### WST-1 cell viability assay

HCC1954 cells were seeded at a number of 2,500 cells/well in 96 well format in 100 μL of 10% FBS medium without antibiotics. After 24 hours of incubation, cells were washed once with PBS and then incubated with 200 μl of either trastuzumab (100 nM) (Roche, Penzberg, Germany) or gefitinib (1 μM) (Biaffin, Kassel, Germany) or c-MYC inhibitor 10058-F4 (80 μM) (Sigma) containing 10% FBS medium. Cells were incubated for 24 hours and each day 20 μl of WST-1 reagent (Roche, Basel) was pipetted to the cells. Absorbance was measured at 450 nm after 2.5 hours with a SpectraMAX 190 (Molecular Devices, UK). The WST-1 assay was performed over four days with one measurement taken on everyday.

### Real-time cell-electrode impedance measurements

One hundred microliters of growth medium was added to the wells of E-plates (Roche, Penzberg) for background measurements. Then, 100 μL of HCC1954, SK-BR-3 and BT474 cell suspensions were added at a number of 8,000 (for HCC1954 cells) and 10000 (for SK-BR-3 and BT474) cells/well. E-plates were incubated at room temperature for 30 minutes; then transferred to the holder and incubated at 37°C with 5% CO_2_. The continuous impedance measurement was recorded and converted to a cell index (CI). After 24 hours, the chemical inhibitors (10058-F4 (80 μM) and gefitinib (1 μM) or/and trastuzumab (100 nM) were added to the respective wells and impedance measurements were continued for 72 hours. Results were analyzed using RTCA Software 1.0 (Roche, Penzberg, Germany).

### Modeling, simulations and data analysis

#### Logical modeling and simulations

A *logical model *is defined by a regulatory graph, where the nodes and arcs represent the regulatory components and interactions, respectively. The dynamical behavior of each component is then defined by logical functions (also represented in terms of logical parameters), which associate a target value for this component depending on the level of its regulators. The dynamics of the system is represented in terms of a state transition graph, where the nodes denote states of the system (*i.e*., a vector giving the levels of activity of all components), and the arcs denote state transitions (*i.e.*, a change in the value of one or several component(s), depending on the values of the relevant logical functions or parameters). In state transition graphs, terminal nodes correspond to "stable states". Note that, for most of the conditions considered, our ERBB receptor regulated G1/S model has a single stable state.

The Boolean model of ERBB signaling network was defined and analyzed using the GINsim software [22,23]. Beginning with relevant initial states, simulations using the logical rules defined in Table 1 was performed. For MOCK case, the initial levels of EGF, all ERBBs, p21 and p27 were set to 1, whereas Cyclin D1 was set to 0. The initial levels of the other proteins were left undefined, meaning that both possible levels were considered. A knockdown can be simulated in GINsim by setting the corresponding protein's initial level and its maximal value to 0. The resulting parameterized model and all simulations can be downloaded from the model repository referred at the GINsim web page: .

#### Analysis of knockdown responses

Statistical significance of protein expression changes and pRB phosphorylation due to knockdowns via RNA interference were calculated using the ANOVA method: *protein expression *~* knockdown effect *+* biological replicate factor *+* error *A multiple testing correction was performed using Benjamini-Hochberg's method [40] with a false discovery rate (FDR) significance cut off of 1%.

#### Network inference

Whenever a knockdown significantly affected the expression of another protein with an FDR < 1%, an edge was drawn. Then a transitive reduction of the graph was calculated (i.e. eliminating putative indirect edges, which could also be explained by another path in the graph [41]. Since the transitive reduction for graphs with cycles is not unique and depends on the ordering of the nodes, we implemented a jackknife procedure, i.e. we left out each node once, estimated the network, and finally counted for each edge the frequency of the occurrence among all jackknife samples. The corresponding jackknife probability is reported at each edge. We also performed the multiple testing corrections separately within each sample of the jackknife procedure, since the false discovery rate depends on the distribution of all raw p-values, which may change with the differing gene selection in each jackknife sample. The R source code is available from the authors upon request. Only the edges having a jackknife probability greater than 50% were kept.

## Abbreviations

Dif-3: Dictyostelium differentiation-inducing factor-3; EGF: Epidermal Growth Factor; FDR: False discovery rate; qRT-PCR: Quantitative real-time polymerase chain reaction; RNAi: RNA interference; siRNA: small interfering RNA; 3-D cell culture: Three dimensional cell culture; 7-AAD: 7-Aminoactinomycin

## Authors' contributions

ÖS, TB and DA designed the research; ÖS, CL, JM and SB performed the research; ÖS, TB, HF, CC and DT carried out computational modeling and simulations; IS and SW participated in sequence analysis; ÖS, HF, CL, UK, MM, CC, DT, TB and DA analyzed data; ÖS, HF, CC, DT, AP, SW, TB and DA wrote the manuscript. All authors read and approved the final manuscript.

## Supplementary Material

Additional file 1 **This folder contains the following items:** 1. Figure 1 and its figure legend (Page 1). 2. Figure 2 and its figure legend (Page 2). 3. Figure 3 and its figure legend (Page 3). 4. Table 1: List of references for Figure 3 (Page 4) 5. Table 2: List of siRNAs and their sequences (Page 5) 6. Table 3: List of antibodies (Page 6). 7. Table 4: List of primers, their sequences and probe numbers (Page 7). Click here for file

Additional file 2 **This folder contains the following items:** 1. Table 1: Stable states and pRB response for single and multiple knockdowns of network proteins (Pages 1–13). 2. Table 2: Analysis of the effects of knockdowns on G1/S transition (p-pRB response) (Page 13). Click here for file
