# Supplementary material for: Modeling ERBB receptor-regulated G1/S transition to find novel targets for de novo trastuzumab resistance
Source: BMC Syst Biol. 2009 Jan 1;3:1. doi: 10.1186/1752-0509-3-1 (PMC2652436; doi:10.1186/1752-0509-3-1)
Supplement: Additional file 1 — This folder contains the following items: 1. Figure 1 and its figure legend (Page 1). 2. Figure 2 and its figure legend (Page 2). 3. Figure 3 and its figure legend (Page 3). 4. Table 1: List of references for Figure 3 (Page 4) 5. Table 2: List of siRNAs and their sequences (Page 5) 6. Table 3: List of antibodies (Page 6). 7. Table 4: List of primers, their sequences and probe numbers (Page 7). [file 1752-0509-3-1-S1.pdf]

ADDITIONAL FILE 1

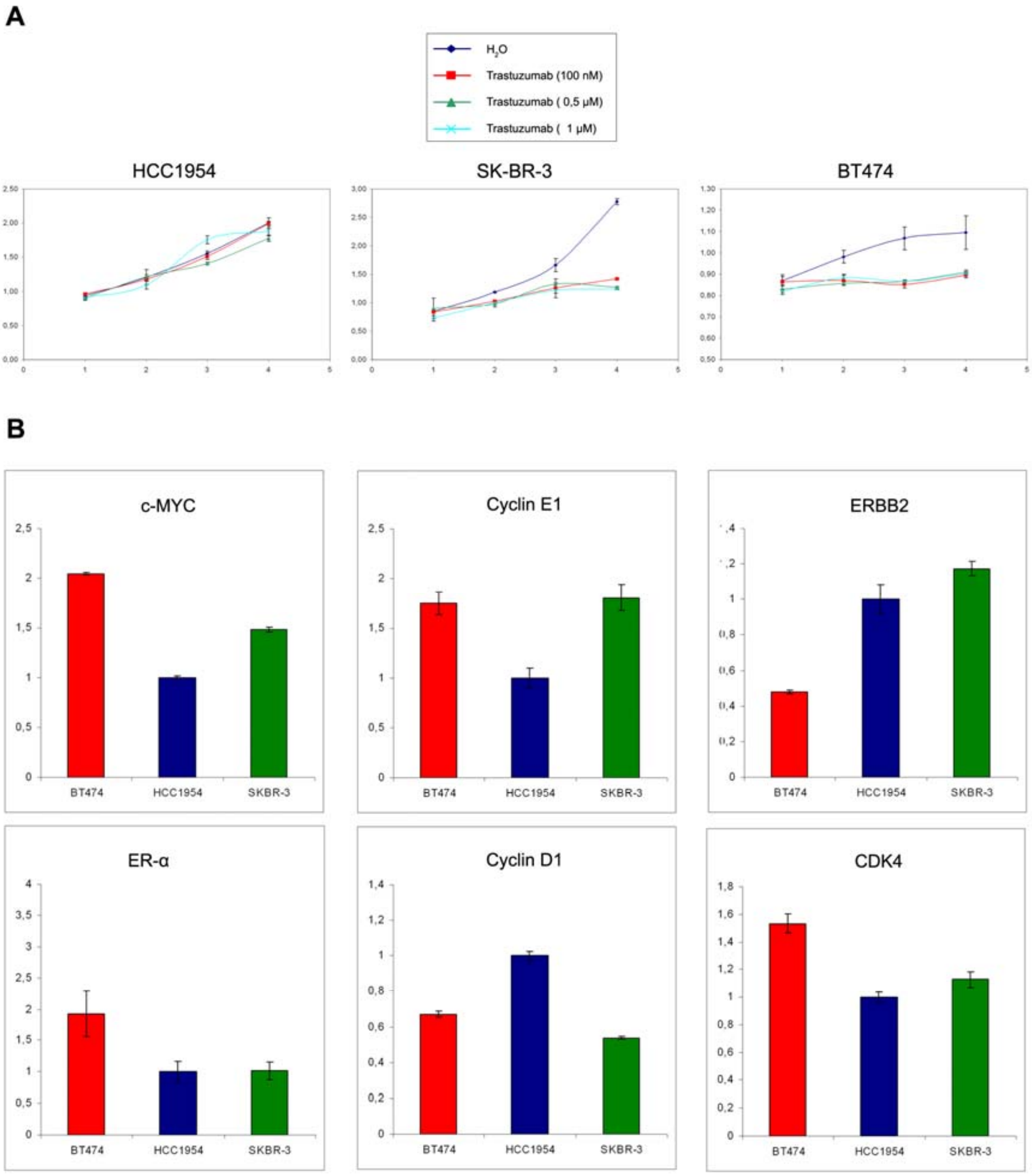

Figure 1. Test of different concentrations of trastuzumab and Reverse Phase Protein Array quantification of network proteins in resistant and sensitive cells. **A.** WST-1 viability assay to determine the response of cells to different trastuzumab concentrations (100 nM, 0.5 µM and 1 µM) over 4 days. Water was used as vehicle control. **B.** Reverse Phase Protein Array results for the quantification of ERBB2

receptor and candidate proteins to be targeted in resistant HCC1954 and sensitive SK-BR-3 and BT474 cells.

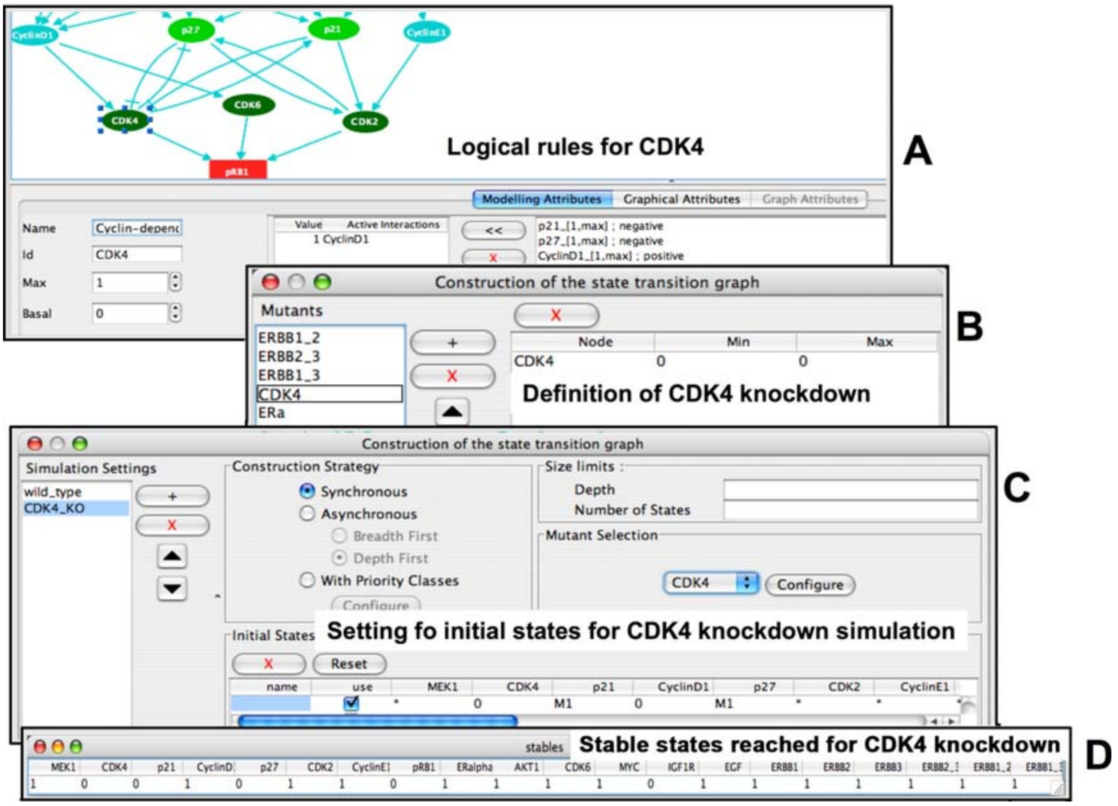

Figure 2. Logical knockdown simulations. **A.** CDK4 knockdown was taken as an example to describe the logical definition of knockdown situations. Logical rule for the activation of CDK4 protein was shown as an example. It shows that CDK4 is activated when Cyclin D1 is active, but neither p21 nor p27. **B.** Minimum and maximum values of CDK4 were set to “0” to implement a knockdown of this factor. **C.** For each knockdown, we determined a set of relevant initial states, with the knockdown proteins set to "0". “0” means inactive, “M1” means active and “*” means that the corresponding protein can take any of these values. **D.** Stable states reached for CDK4 knockdown, with pRB = 0.

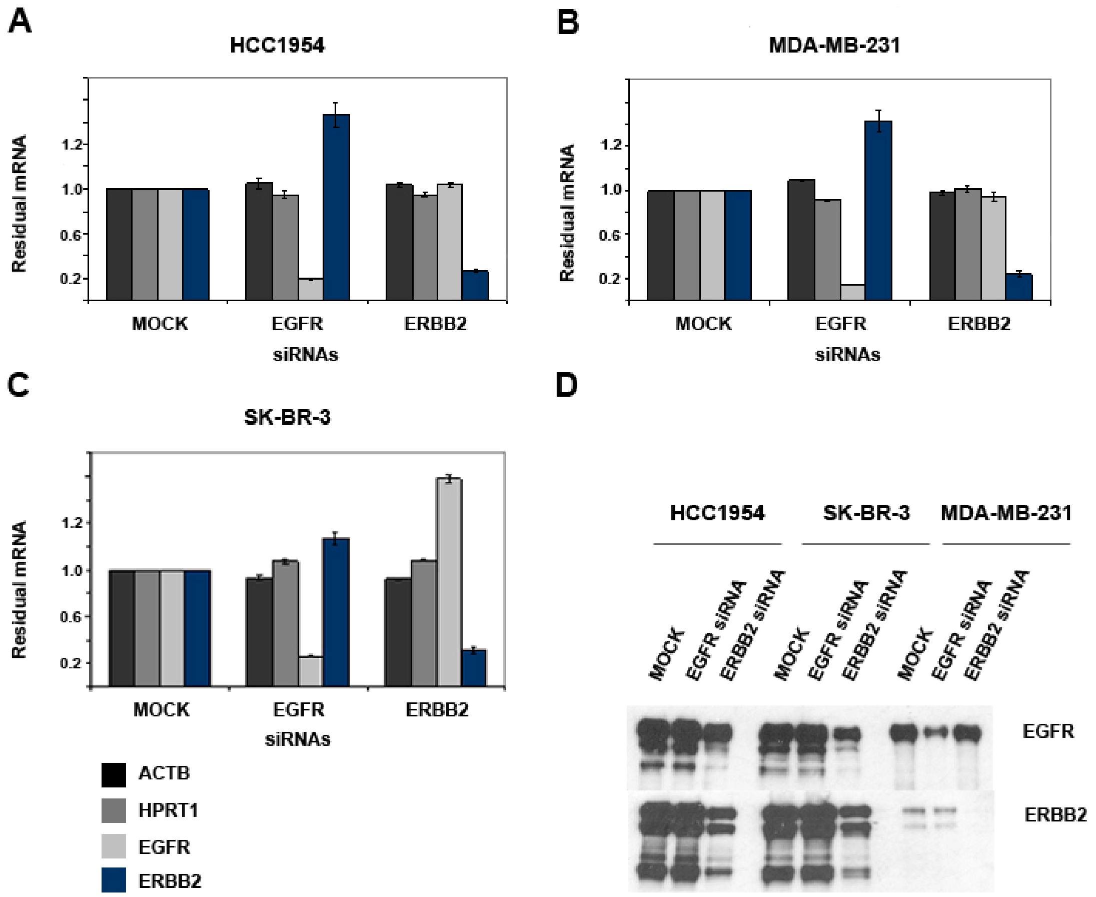

Figure 3. Stabilization of EGFR protein by ERBB2 in ERBB2 overexpressing HCC1954 and SK-BR-3 cells, but not in low ERBB2 expressing MDA-MB-231 cells. **A, B** and **C.** qRT-PCR results showing the knockdown efficiencies of EGFR and ERBB2 siRNA pools (20 nM) at mRNA level in HCC1954, MDA-MB-231 and SK_BR_3 cells, respectively. ACTB and HPRT1 genes were used as house-keeping controls. **D.** Western blots demonstrating the knockdown efficiencies of EGFR and ERBB2 siRNAs at protein level in ERBB2 overexpressing HCC1954 and SK-BR-3 cells, and in low ERBB2 expressing MDA-MB-231 cells. In ERBB2 siRNA transfected cells both EGFR and ERBB2 were down regulated in HCC1954 and SK-BR-3 cells, but not in MDA-MB-231 cells although siRNAs have no targeting activity against each other at mRNA level (A, B and C).

Table 1 The list of references for each interaction shown in Figure 3

1	EGF binds to ERBB1 homodimer and this results in its activation	Holbro T, et al, 2003 (Experimental Cell Research)
2	EGF stimulates ERBB1/ERBB2 heterodimer and results in phosphorylation of Ty1248 residue of ErbB2	Holbro T, et al, 2003 (Experimental Cell Research) / Johannessen LE et al, 2001 (Biochem. J)
3	EGF binds to the EGFR and promotes the formation of EGFR/EGFR homodimers and EGFR/ERBB2 heterodimers and, less frequently, EGFR/ERBB3	Anido J et al, 2003 (Clinical Cancer Research) / Alroy I and Yarden Y, 1997 (Febs Lett.)
4	EGF stimulates the formation of ERBB2/ERBB3 hetero-oligomers in cells that also express the EGFR	Huang GC and Ouyang RJ, 1998 (Biochem. J) / Gamett DC et al, 1997 (J. Biol. Chem.)
5	ERBB2/ERBB3 protein decreases expression of IGF1R mRNA	Alaoui-Jamali MA et al, 2003 (Cancer Research)
6	The docking protein Gab1 is the primary mediator of EGF-stimulated activation of the PI-3K/Akt cell survival pathway (EGFR do not include canonical PI-3 kinase binding sites)	Mattoon DR, et al, 2004 (BMC Biol.)
7	Following EGFR phosphorylation, Ras-GTP activates Raf-1 that phosphorylates MAPK	Hallberg B et al, 1994 (J Biol Chem) / Liebmann C, 2001 (Cell signal)
8	ERBB1/ERBB2 heterodimers are potent in activating the phosphoinositide 3'-kinase (PI3K)	Zhan L et al, 2006 (Cancer Research)
9	ERBB1/ERBB2 heterodimers are potent in activating the Ras/mitogen-activated protein kinase pathway	Zhan L et al, 2006 (Cancer Research)
10	The principal mechanism that drives EGFR-dependent PI3K activation is the dimerization of the receptor with ERBB3	Scaltriti M and Baselga J, 2006 (Clinical Cancer Research)
11	ERBB1/ERBB2 heterodimers can activate MAPK pathway by docking Grb2/Ras proteins	Alroy I and Yarden Y, 1997 (FEBS Lett)
12	ERBB3 couple active ERBB2 to the phosphatidylinositol 3-kinase/protein kinase B pathway	Holbro T et al, 2003 (PNAS)
13	High levels ERBB2/ERBB3 signalling lead to persistent Ras activity via amplification of MAPK pathway	Butt AJ et al, 2005 (Endocrine-Related Cancer)
14	IGF1R activates docking protein IRS-1 which is responsible for coupling to PI3K pathway	Shelton JG et al, 2004 (Cell Cycle)
15	IGF1R activates docking substrate Shc which activates SOS, Ras and downstream Raf/MEK/ERK cascade	Shelton JG et al, 2004 (Cell Cycle)
16	AKT1 protein increases expression of human IGF1R protein.	Tanno S et al, 2001 (Cancer Research)
17	ER alpha protein increases expression/phosphorylation of IGF1R protein.	Levin ER, 2003 (Mol Endocrinnology) / Oesterreich S et al, 2001 (Cancer Research)
18	Activation of PI3K/AKT results in ligand-independent activation of ER alpha	Martin MB et al, 2000 (Endocrinology)
19	Inhibition of PI3K by LY294002 decreases the half-life of the 4.5 kb Cyclin D1 mRNA / AKT stabilizes Cyclin D	Dufourny B et al, 2000 (J. Endocrinology)
20	AKT phosphorlates p27 on residues within its nuclear localiazation motifs leading to relocalization to cytoplasm (no inhibiton of Cyclin E/CDK2 complex)	Liang J and Slingerland JM, 2003 (Cell Cycle)
21	AKT phosphorlates p21 on residues within its nuclear localiazation motifs leading to relocalization to cytoplasm (no inhibiton of Cyclin E/CDK2 complex)	Liang J and Slingerland JM, 2003 (Cell Cycle)
22	PI3K/AKT pathway stimulates translation of C-MYC mRNA and stabilization of the protein	Sears R et al, 2000 (Genes Dev.)
23	(MAPK) activated by growth factors phosphorylates at Ser 118 and potentiates the N-terminal transactivation function (AF-1)	Kato S et al, 1995 (Science)
24	Sustained ERK activity increases Cyclin D1 mRNA and protein level / Raf/MEK/ERK cascade increases transcription of cyclin D1	Weber JD et al, 1997 (Biochem J)/Coleman et al, 2004
25	Ras/Raf/MEK/MAPK pathway regulates C-MYC expression	Cheng M et al, 1999 (J Biol Chem)/Sears R et al, 2000 (Genes Dev.)
26	ER activation at the Cyclin D1 promoter is mediated by both the cyclic AMP-response element and the activating protein-1 site	Liu MM et al, 2002 (J Biol Chem)
27	Adenoviral expression of ER alpha in ER-negative breast cancer cells leads to increase in protein levels p27 cyclin-dependent kinase inhibitor	Licznar A et al, 2003 (Febs Lett)
28	ER alpha induced the expression of several endogenous genes such as the cyclin kinase inhibitor p21	Lazennec et al, 2001 (Endocrinology) / Zhao H et al 2004 (J. Cell Biochem.)
29	Induction of C-MYC through ER alpha leads to activation of Cyclin E/CDK2 complex via reduction of p21	Sutherland RL et al, 1998 (J Mammary Gland Biol Neoplasia)
30	Cyclin D1 expression is regulated by C-MYC at transcriptional level	Bartek J and Lukas J, 2001 (FEBS Letters)
31	MYC acts as a upstream regulator of CDKs and functionally antagonizes the action of p27	Maddika S et al, 2007 (Drug Resistance Updates)
32	Interaction of MYC-MAX heterodimer with MIZ-1 and/or SP-1 inactivates p21	Pelengaris et al, 2002 (Nature Reviews Cancer)
33	Induction of C-MYC leads to activation of Cyclin E/CDK2 complex via reduction of p21	Prall OW et al, 1998 (MCB)
34	Cyclin D1 binds to CDK4 to activate its kinase activity	Morgan DO, 1997 (Ann Rev Cell Dev Biol)
35	Cyclin D1 binds to CDK6 to activate its kinase activity	Vermeulen K et al, 2003 (Cell Prolif.)
36	p27 inhibits the activity of CDK4	Cariou S et al, 2000 (PNAS)
37	The growth factor activation of Cyclin D/CDK4 complex sequesters unbound p27 and inhibits its inhiborty effect of Cyclin E/CDK2 complex	Grillo M et al, 2006 (Breast Cancer Research and Treatment)
38	The cyclin E-CDK2 complex positively regulates its activity by phosphotylating p27, which is then targeted for degradation	Sherr CJ and Roberts JM, 2004 (Gene Dev.)/ Müller D et al, 1997 (Oncogene)
39	p27 prevents the activation of CDK2	Cariou S et al, 2000 (PNAS)
40	The growth factor activation of Cyclin D/CDK4 complex sequesters unbound p21 and inhibits its inhiborty effect of Cyclin E/CDK2 complex	Grillo M et al, 2006 (Breast Cancer Research and Treatment)
41	p21 inhibits the activity of CDK4	Harper JW et al, 1995 (MBC)
42	p21 inhibits the activity of CDK2	Harper JW et al, 1995 (MBC)
43	Cyclin D/CDK4 complex phosphorylates pRB protein at Ser 807/811	Zarkowska T et al, 1997 (J Biol Chem)
44	Activated cyclin D/CDK4 or CDK6 complex phosphorylates pRb, releasing it from its growth-suppressive functions	Yu B et al, 2000 (Molecular Cell Biology Research Communications)
45	Cyclin E binds to and activates CDK2 to regulate G1/S transition	Ohtsubo M et al, 1995 (Mol Cell Biol)
46	The activated cyclin E/CDK2 complex also contributes to the phosphorylation of pRB late in G1	Hatakeyama M, 1994 (Genes Dev) / Hinds P et al, 1994 (PNAS)

Table 2 The list of siRNAs and sequences

Gene	Accession number	No.	Sense Sequence	Antisense Sequence
MAP2K1	NM_002755	1	GCACAUGGAUGGAGGUUCUUU	5'-P AGAACCUCCSUCCSUGUGCUU
MAP2K1	NM_002755	2	GCAGAGAGAGCAGAUUUGAUU	5'-P UCAAAUCUGCUCUCUCUGCUU
MAP2K1	NM_002755	3	GAGCAGAUUUGAAGCAACUUU	5'-P AGUUGCUUCAAAUCUGCUCUU
MAP2K1	NM_002755	4	CCAGAAAGCUAAUUCAUCUUU	5'-P AGAUGAAUUAGCUUUCUGGUU
AKT1	NM_005163	1	GACAAGGACGGGCACAUUAUU	5'-P UAAUGUGCCCGUCCUUGUCUU
AKT1	NM_005163	2	GGACAAGGACGGGCACAUUUU	5'-P AAUGUGCCCGUCCUUGUCCUU
AKT1	NM_005163	3	GCUACUUCCUCCUCAAGAAUU	5'-P UUCUUGAGGAGGAAGUAGCUU
AKT1	NM_005163	4	GACCGCCUCUGCUUUGUCAUU	5'-P UGACAAAGCAGAGGCGGUCUU
ESR1	NM_000125	1	GAAUGUGCCUGGCUAGAGAUU	5'-P UCUCUAGCCAGGCACAUUCUU
ESR1	NM_000125	2	CAUGAGAGCUGCCAACCUUUU	5'-P AAGGUUGGCAGCUCUCAUGUU
ESR1	NM_000125	3	AGAGAAAGAUUGGCCAGUAUU	5'-P UACUGGCCAAUCUUUCUCUUU
ESR1	NM_000125	4	GAUCAAACGCUCUAAGAAGUU	5'-P CUUCUUAGAGCGUUUGAUCUU
CDKN1B	NM_004064	1	GGAGCAAUGCGCAGGAAUAUU	5'-P UAUUCCUGCGCAUUGCUCCUU
CDKN1B	NM_004064	2	GAGCAAUGCGCAGGAAUAAUU	5'-P UUWUUCCUGCGCSUUGCUCUU
CDKN1B	NM_004064	3	GCAAUGCGCAGGAAUAAGGUU	5'-P CCUUAUUCCUGCGCAUUGCUU
CDKN1B	NM_004064	4	CGACGAUUCUUCUACUCAAUU	5'-P UUGAGUAGAAGAAUDGUDGUU
CDKN1A	NM_000389	1	GAUGGAACUUCGACUUUGUUU	5'-P ACAAAGUCGAAGUUCCAUCUU
CDKN1A	NM_000389	2	GCGAUGGAACUUCGACUUUUU	5'-P AAAGUCGAAGUUCCAUCGCUU
CDKN1A	NM_000389	3	CGAUGGAACGGCGACUUUGUU	5'-P CAAAGUCGAAGUUCCAUCGUU
CDKN1A	NM_000389	4	CGACUGUGAUGCGCUAAUGUU	5'-P CAUUAGCGCAUCACAGUCGUU
CDK4	NM_000075	1	GCAGCACUCUUAUCUACAUUU	5'-P AUGUAGAUAAGAGUGCUGCUU
CDK4	NM_000075	2	GGAGGAGGCCUUCCCAUCAUU	5'-P UGAUGGGAAGGCCUCCUCCUU
CDK4	NM_000075	3	UCGAAAGCCUCUCUUCUGUUU	5'-P ACAGAAGAGAGGCUUUCGAUU
CDK4	NM_000075	4	GUACCGAGCUCCCGAAGUUUU	5'-P AACUUCGGGAGCUCGGUACUU
CDK2	NM_001798	1	GAGCUAACCAUCCUAAUAUU	5'-P UAUUAGGAUGGUUAAGCUCUU
CDK2	NM_001798	2	GAGAGGUGGUGCGCUUAAUU	5'-P UUAAGCGCCACCACCUCUCUU
CDK2	NM_001798	3	GCACCAAGAUCUCAAGAAAU	5'-P UUUCUUGAGAUCUUGGUGCUU
CDK2	NM_001798	4	GGACGGAGCUUGUUAUCGCUU	5'-P GCGAUAACAAGCUCCGUCCUU
CCNE1	NM_001238	1	GGAAAUCUAUCCUCCAAAGUU	5'-P CUUUGGAGGAUAGAUUUCCUU
CCNE1	NM_001238	2	GGAGGUGUGUGAAGUCUAUUU	5'-P AUAGACUUCACACACCUCCUU
CCNE1	NM_001238	3	CUAAAUGACUUACAUGAAGUU	5'-P CUUCAUGUAAGUCAUUUCGUU
CCNE1	NM_001238	4	GUAUAUGGCGACACAAGAAUU	5'-P UUCUUGUGUCGCCAUAUACUU
CCND1	NM_053056	1	GUUCGUGGCCUCUAAGAUGUU	5'-P CAUCUUAGAGGCCACGAACUU
CCND1	NM_053056	2	CCGAGAAGCUGUGCAUCUAUU	5'-P UAGAUGCACAGCUUCUCGGUU
CCND1	NM_053056	3	GAACAGAAGUGCGAGGAGGUU	5'-P CCUCCUCGCACUUCUGUUCUU
CCND1	NM_053056	4	ACAACUUCCUGUCCUACUAUU	5'-P UAGUAGGACAGGAAGUUGUUU
IGF1R	NM_000875	1	GGCCAGAAAUGGAGAAUAAUU	5'-P UUAUUCUCCAUUUCUGGCCUU
IGF1R	NM_000875	2	GCAGACACCUACAACAUCAUU	5'-P UGAUGUUGUAGGUGUCUGCUU
IGF1R	NM_000875	3	GGACUCAGUACGCCGUUUAUU	5'-P UAAACGGCGUACUGAGUCCUU
IGF1R	NM_000875	4	GUGGGAGGGUUGGUGAUUAUU	5'-P UAAUCACCAACCCUCCCACUU
MYC	NM_002467	1	ACGGAACUCUUGUGCGUAAUU	5'-P UUACGCACAAGAGUUCCGUUU
MYC	NM_002467	2	GAACACACAACGUCUUGGAUU	5'-P UCCAAGACGUUGUGUGUUCUU
MYC	NM_002467	3	AACGUUAGCUUCACCAACAUU	5'-P UGUUGGUGAAGCUAACGUUUU
MYC	NM_002467	4	CGAUGUUGUUUCUGUGGAAUU	5'-P UUCCACAGAAACAACAUCGUU
ERBB3	NM_001005915	1	GCGAUGCUGAGAACCAAUAUU	5'-P UAUUGGUUCUCAGCAUCGCUU
ERBB3	NM_001005915	2	AGAUUGUGCUCACGGGACAUU	5'-P UGUCCCGUGAGCACAAUCUUU
ERBB3	NM_001005915	3	GCAGUGGAUUCGAGAAGUGUU	5'-P CACUUCUCGAAUCCACUGCUU
ERBB3	NM_001005915	4	UCGUCAUGUUGAACUAUAAUU	5'-P UUAUAGUUCAACAUGACGAUU
ERBB2	NM_004448	1	UGGAAGAGAUCACAGGUUAUU	5'-P UAACCUGUGAUCUCUUCCAUU
ERBB2	NM_004448	2	GAGACCCGCUGAACAAUACUU	5'-P GUAUUGUUCAGCGGGUCUCUU
ERBB2	NM_004448	3	GGAGGAAUGCCGAGUACUGUU	5'-P CAGUACUCGGCAUUCCUCCUU
ERBB2	NM_004448	4	GCUCAUCGCUCACAACCAAUU	5'-P UUGGUUGUGAGCGAUGAGCUU
EGFR	NM_201283	1	CAAAGUGUGUAACGGAAUAUU	5'-P UAUUCCGUUACACACUUUGUU
EGFR	NM_201283	2	CCAUAAAUGCUACGAAUAUUU	5'-P AUAUUCGUAGCAUUUAUGGUU
EGFR	NM_201283	3	GUAACAAGCUCACGCAGUUUU	5'-P AACUGCGUGAGCUUGUUACUU
EGFR	NM_201283	4	CAGAGGAUGUUCAAUAACUUU	5'-P AGUUAUUGAACAUCCUCUGUU
CDK6	NM_001259	1	GCAAAGACCUACUUCUGAAUU	5'-P UUCAGAAGUAGGUCUUUGCUU
CDK6	NM_001259	2	UAACAGAUAUCGAUGAACUUU	5'-P AGUUCAUCGAUAUCUGUUAUU
CDK6	NM_001259	3	GAUAUGAUGUUUCAGCUUCUU	5'-P GAAGCUGAAACAUCAUAUCUU
CDK6	NM_001259	4	GCACUAAUCAGCACACAUAUU	5'-P UAUGUGUGCUGAUUAGUGCUU

Table 3 The list of antibodies

Antibody	Company	Catalog no	Dilution WB	Dilution RPPA	Storage (C)	Species
EGFR	Santa Cruz	sc-03	1/500	1/500	4	Rabbit
ERBB2	Neomarkers	Ab-17	1/500	1/500	4	Mouse
ERBB3	Upstate	05-390	1/500	NA	-20	Mouse
ERBB4	Upstate	06-572	1/300	NA	-20	Rabbit
IGF1R	Upstate	05-656	1/500	NA	-20	Mouse
ER alpha	Upstate	05-820	1/250	NA	-20	Rabbit
MYC	Cell Signalling Technolgies	9402	1/500	NA	-20	Rabbit
AKT1	BD	610860	1/1000	1/500	-20	Mouse
pAKT1	Covance	PRB-542P	1/1000	1/250	-20	Rabbit
MEK1	BD	610122	1/1000	NA	-20	Mouse
ERK1/2	Santa Cruz	sc-94(K-23)	1/1000	1/500	4	Rabbit
pERK1/2	RD systems	AF1018	1/1000	1/500	-20	Rabbit
p21	BD	610233	1/500	1/200	-20	Mouse
p27	BD	610241	1/1000	1/200	-20	Mouse
CDK2	BD	610146	1/500	1/200	-20	Mouse
CDK4	Cell Signalling Technolgies	2906	1/500	1/200	-20	Mouse
CDK6	Sigma	C8343	1/500	NA	4	Mouse
Cyclin D1	Cell Signalling Technolgies	2926	1/500	1/200	-20	Mouse
Cyclin E1	Santa Cruz	sc-247	1/500	NA	4	Mouse
pRB	Cell Signalling Technolgies	9308	1/500	1/250	-20	Rabbit

Table 4 The list of primers, primer sequences and probe numbers

Gene	Gene ID	UPL Probe #	UPL Probe Catalog #	Primer sequence
EGFR_left	NM_005228.3	50	4688112001	acacagaatctatacccaccagagt
EGFR_right	NM_005228.3	50	4688112001	atcaactcccaaacggtcac
ERBB2_left	NM_004448.2	4	4685016001	gggaaacctggaactcacct
ERBB2_right	NM_004448.2	4	4685016001	ccctgcacctcctggata
ERBB3_left	NM_001982.2	86	4689119001	cacaatgccgacctctcc
ERBB3_right	NM_001982.2	86	4689119001	cacgaggacatagcctgtca
IGF1R_left	NM_000875.2	55	4688520001	aaaaaccttcgcctcatcc
IGF1R_right	NM_000875.2	55	4688520001	tggttgtcgaggacgtagaa
ESR1_left	NM_000125.2	24	4686985001	ttactgaccaacctggcaga
ESR1_right	NM_000125.2	24	4686985001	atcatggagggtcaaatcca
MYC_left	NM_002467.3	75	4688988001	tttttcgggtagtggaaaacc
MYC_right	NM_002467.3	75	4688988001	ttcctgttggtgaagctaacg
AKT1_left	NM_005163.2	45	4688058001	gcagcacgtgtacgagaaga
AKT1_right	NM_005163.2	45	4688058001	ggtgtcagtctccgacgtg
MAP2K1_left	NM_002755.2	7	4685059001	ttttaggaaaagttagcattgctgt
MAP2K1_right	NM_002755.2	7	4685059001	agggcttgacatctctgtgc
CDKN1A_left	NM_078467.1	32	4687655001	tcactgtcttgtacccttgtgc
CDKN1A_right	NM_078467.1	32	4687655001	ggcgtttggagtggtagaaa
CDKN1B_left	NM_004064.2	1	4684974001	agatgtcaaacgtgcgagtg
CDKN1B_right	NM_004064.2	1	4684974001	cgggttaactcttcgtggtc
CCND1_left	NM_053056.2	67	4688660001	gaagatcgtcgccacctg
CCND1_right	NM_053056.2	67	4688660001	gacctcctcctcgcacttct
CCNE1_left	NM_001238.1	36	4687949001	ggccaaaatcgacaggac
CCNE1_right	NM_001238.1	36	4687949001	gggtctgcacagactgcat
CDK2_left	NM_001798.2	50	4688112001	cctcctgggctgcaaata
CDK2_right	NM_001798.2	50	4688112001	cagaatctccagggaataggg
CDK4_left	NM_000075.2	25	4686993001	gtgcagtcggtggtacctg
CDK4_right	NM_000075.2	25	4686993001	tgtgtgggttaaaagtcagca
CDK6_left	NM_001259.5	2	4684982001	tgatcaactaggaaaaatcttggac
CDK6_right	NM_001259.5	2	4684982001	ggcaacatctctaggccagt
