# Supplementary material for: Modeling ERBB receptor-regulated G1/S transition to find novel targets for de novo trastuzumab resistance
Source: BMC Syst Biol. 2009 Jan 1;3:1. doi: 10.1186/1752-0509-3-1 (PMC2652436; doi:10.1186/1752-0509-3-1)
Supplement: Additional file 2 — This folder contains the following items: 1. Table 1: Stable states and pRB response for single and multiple knockdowns of network proteins (Pages 1–13). 2. Table 2: Analysis of the effects of knockdowns on G1/S transition (p-pRB response) (Page 13). [file 1752-0509-3-1-S2.pdf]

ADDITIONAL FILE 2

Table 1 Stable states and pRB response for single and multiple knockdowns of network proteins. Green rows demonstrate the stable states obtained for knockdown simulations, which are the same as MOCK (unperturbed) case. Grey rows indicate the knockdown simulations, for which there are two stable states. pRB1 in red shows that the corresponding knockdown simulations result in pRB=1 (i.e. there is G1/S transition).

Knockdowns	Gene	Min	Max	Stable State(s)
MOCK	-	-	-	**1 Stable state**
				MEK1 ; CDK4 ; CyclinD1 ; CDK2 ; CyclinE1 ; **pRB1** ; ERalpha ; AKT1 ; CDK6 ; MYC ; EGF ; ERBB1 ; ERBB2 ; ERBB3 ; ERBB2_3 ; ERBB1_2 ; ERBB1_3
AKT1	AKT1	0	0	**1 Stable state**
				MEK1 ; CDK4 ; CyclinD1 ; CDK2 ; CyclinE1 ; **pRB1** ; ERalpha ; CDK6 ; MYC ; EGF ; ERBB1 ; ERBB2 ; ERBB3 ; ERBB2_3 ; ERBB1_2 ; ERBB1_3
MEK1	MEK1	0	0	**1 Stable state**
				CDK4 ; CyclinD1 ; CDK2 ; CyclinE1 ; **pRB1** ; ERalpha ; AKT1 ; CDK6 ; MYC ; EGF ; ERBB1 ; ERBB2 ; ERBB3 ; ERBB2_3 ; ERBB1_2 ; ERBB1_3
CDK2	CDK2	0	0	**1 Stable state**
				MEK1 ; CDK4 ; CyclinD1 ; CyclinE1 ; **pRB1** ; ERalpha ; AKT1 ; CDK6 ; MYC ; EGF ; ERBB1 ; ERBB2 ; ERBB3 ; ERBB2_3 ; ERBB1_2 ; ERBB1_3
CDK4	CDK4	0	0	**1 Stable state**
				MEK1 ; CyclinD1 ; CDK2 ; CyclinE1 ; ERalpha ; AKT1 ; CDK6 ; MYC ; EGF ; ERBB1 ; ERBB2 ; ERBB3 ; ERBB2_3 ; ERBB1_2 ; ERBB1_3
p21	p21	0	0	**1 Stable state**
				MEK1 ; CDK4 ; CyclinD1 ; CDK2 ; CyclinE1 ; **pRB1** ; ERalpha ; AKT1 ; CDK6 ; MYC ; EGF ; ERBB1 ; ERBB2 ; ERBB3 ; ERBB2_3 ; ERBB1_2 ; ERBB1_3
p27	p27	0	0	**1 Stable state**
				MEK1 ; CDK4 ; CyclinD1 ; CDK2 ; CyclinE1 ; **pRB1** ; ERalpha ; AKT1 ; CDK6 ; MYC ; EGF ; ERBB1 ; ERBB2 ; ERBB3 ; ERBB2_3 ; ERBB1_2 ; ERBB1_3
CycD1	CyclinD1	0	0	**1 Stable state**
				MEK1 ; CDK2 ; CyclinE1 ; ERalpha ; AKT1 ; MYC ; EGF ; ERBB1 ; ERBB2 ; ERBB3 ; ERBB2_3 ; ERBB1_2 ; ERBB1_3
ERBB2_3	ERBB2	0	0	**1 Stable state**
	ERBB3	0	0	
				MEK1 ; CDK4 ; CyclinD1 ; CDK2 ; CyclinE1 ; **pRB1** ; ERalpha ; AKT1 ; CDK6 ; MYC ; IGF1R ; EGF ; ERBB1
ERBB1_3	ERBB1_3	0	0	**2 Stable states**
	ERBB1	0	0	
	ERBB3	0	0	
				p21 ; p27 ; EGF ; ERBB2

	MEK1 ; CDK4 ; CyclinD1 ; CDK2 ; CyclinE1 ; **pRB1** ; ERalpha ; AKT1 ; CDK6 ; MYC ; IGF1R ; EGF ; ERBB2			
ERBB1	ERBB1	0	0	**1 Stable state**
	MEK1 ; CDK4 ; CyclinD1 ; CDK2 ; CyclinE1 ; **pRB1** ; ERalpha ; AKT1 ; CDK6 ; MYC ; EGF ; ERBB2 ; ERBB3 ; ERBB2_3			
ERBB1_2	ERBB1_2	0	0	**2 Stable states**
	ERBB1	0	0	
	ERBB2	0	0	
	p21 ; p27 ; EGF ; ERBB3			
	MEK1 ; CDK4 ; CyclinD1 ; CDK2 ; CyclinE1 ; **pRB1** ; ERalpha ; AKT1 ; CDK6 ; MYC ; IGF1R ; EGF ; ERBB3			
IGF1R	IGF1R	0	0	**1 Stable state**
	MEK1 ; CDK4 ; CyclinD1 ; CDK2 ; CyclinE1 ; **pRB1** ; ERalpha ; AKT1 ; CDK6 ; MYC ; EGF ; ERBB1 ; ERBB2 ; ERBB3 ; ERBB2_3 ; ERBB1_2 ; ERBB1_3			
CDK6	CDK6	0	0	**1 Stable state**
	MEK1 ; CDK4 ; CyclinD1 ; CDK2 ; CyclinE1 ; ERalpha ; AKT1 ; MYC ; EGF ; ERBB1 ; ERBB2 ; ERBB3 ; ERBB2_3 ; ERBB1_2 ; ERBB1_3			
ERa	ERalpha	0	0	**1 Stable state**
	MEK1 ; CDK2 ; CyclinE1 ; AKT1 ; MYC ; EGF ; ERBB1 ; ERBB2 ; ERBB3 ; ERBB2_3 ; ERBB1_2 ; ERBB1_3			
MYC	MYC	0	0	**1 Stable state**
	MEK1 ; ERalpha ; AKT1 ; EGF ; ERBB1 ; ERBB2 ; ERBB3 ; ERBB2_3 ; ERBB1_2 ; ERBB1_3			
CyclinE1	CyclinE1	0	0	**1 Stable state**
	MEK1 ; CDK4 ; CyclinD1 ; **pRB1** ; ERalpha ; AKT1 ; CDK6 ; MYC ; EGF ; ERBB1 ; ERBB2 ; ERBB3 ; ERBB2_3 ; ERBB1_2 ; ERBB1_3			
ERBB2	ERBB2	0	0	**1 Stable state**
	MEK1 ; CDK4 ; CyclinD1 ; CDK2 ; CyclinE1 ; **pRB1** ; ERalpha ; AKT1 ; CDK6 ; MYC ; IGF1R ; EGF ; ERBB1 ; ERBB3 ; ERBB1_3			
ERRB2_MYC	MYC	0	0	**1 Stable state**
	ERBB2	0	0	
	MEK1 ; ERalpha ; AKT1 ; IGF1R ; EGF ; ERBB1 ; ERBB3 ; ERBB1_3			
EGF	EGF	0	0	**2 Stable states**
	p21 ; p27			
	MEK1 ; CDK4 ; CyclinD1 ; CDK2 ; CyclinE1 ; **pRB1** ; ERalpha ; AKT1 ; CDK6 ; MYC ; IGF1R			
AKT1_MEK1	AKT1	0	0	**1 Stable state**
	MEK1	0	0	
	p21 ; p27 ; EGF ; ERBB1 ; ERBB2 ; ERBB3 ; ERBB2_3 ; ERBB1_2 ; ERBB1_3			
AKT1_CDK2	AKT1	0	0	**1 Stable state**
	CDK2	0	0	
	MEK1 ; CDK4 ; CyclinD1 ; CyclinE1 ; **pRB1** ; ERalpha ; CDK6 ; MYC ; EGF ; ERBB1 ; ERBB2 ; ERBB3 ; ERBB2_3 ; ERBB1_2 ; ERBB1_3			
AKT1_CDK4	AKT1	0	0	**1 Stable state**
	CDK4	0	0	
	MEK1 ; CyclinD1 ; CDK2 ; CyclinE1 ; ERalpha ; CDK6 ; MYC ; EGF ; ERBB1 ; ERBB2 ; ERBB3 ; ERBB2_3 ; ERBB1_2 ; ERBB1_3			
AKT1_p21	AKT1	0	0	**1 Stable state**

	MEK1 ; CDK4 ; CyclinD1 ; CDK2 ; CyclinE1 ; **pRB1** ; ERalpha ; CDK6 ; MYC ; EGF ; ERBB1 ; ERBB2 ; ERBB3 ; ERBB2_3 ; ERBB1_2 ; ERBB1_3			
AKT1_p27	AKT1	0	0	**1 Stable state**
	p27	0	0	
	MEK1 ; CDK4 ; CyclinD1 ; CDK2 ; CyclinE1 ; **pRB1** ; ERalpha ; CDK6 ; MYC ; EGF ; ERBB1 ; ERBB2 ; ERBB3 ; ERBB2_3 ; ERBB1_2 ; ERBB1_3			
AKT1_CycD1	AKT1	0	0	**1 Stable state**
	CyclinD1	0	0	
	MEK1 ; CDK2 ; CyclinE1 ; ERalpha ; MYC ; EGF ; ERBB1 ; ERBB2 ; ERBB3 ; ERBB2_3 ; ERBB1_2 ; ERBB1_3			
AKT1_ERBB2_3	AKT1	0	0	**1 Stable state**
	ERBB2	0	0	
	ERBB3	0	0	
	MEK1 ; CDK4 ; CyclinD1 ; CDK2 ; CyclinE1 ; **pRB1** ; ERalpha ; CDK6 ; MYC ; IGF1R ; EGF ; ERBB1			
AKT1_ERBB1_3	AKT1	0	0	**2 Stable states**
	ERBB1_3	0	0	
	ERBB1	0	0	
	ERBB3	0	0	
	p21 ; p27 ; EGF ; ERBB2			
	MEK1 ; CDK4 ; CyclinD1 ; CDK2 ; CyclinE1 ; **pRB1** ; ERalpha ; CDK6 ; MYC ; IGF1R ; EGF ; ERBB2			
AKT1_ERBB1	AKT1	0	0	**1 Stable state**
	ERBB1	0	0	
	MEK1 ; CDK4 ; CyclinD1 ; CDK2 ; CyclinE1 ; **pRB1** ; ERalpha ; CDK6 ; MYC ; EGF ; ERBB2 ; ERBB3 ; ERBB2_3			
AKT1_ERBB2	AKT1	0	0	**1 Stable state**
	ERBB2	0	0	
	MEK1 ; CDK4 ; CyclinD1 ; CDK2 ; CyclinE1 ; **pRB1** ; ERalpha ; CDK6 ; MYC ; IGF1R ; EGF ; ERBB1 ; ERBB3 ; ERBB1_3			
AKT1_ERBB1_2	AKT1	0	0	**2 Stable states**
	ERBB1_2	0	0	
	ERBB1	0	0	
	ERBB2	0	0	
	p21 ; p27 ; EGF ; ERBB3			
	MEK1 ; CDK4 ; CyclinD1 ; CDK2 ; CyclinE1 ; **pRB1** ; ERalpha ; CDK6 ; MYC ; IGF1R ; EGF ; ERBB3			
AKT1_IGF1R	AKT1	0	0	**1 Stable state**
	IGF1R	0	0	
	MEK1 ; CDK4 ; CyclinD1 ; CDK2 ; CyclinE1 ; **pRB1** ; ERalpha ; CDK6 ; MYC ; EGF ; ERBB1 ; ERBB2 ; ERBB3 ; ERBB2_3 ; ERBB1_2 ; ERBB1_3			
AKT1_CDK6	AKT1	0	0	**1 Stable state**
	CDK6	0	0	
	MEK1 ; CDK4 ; CyclinD1 ; CDK2 ; CyclinE1 ; ERalpha ; MYC ; EGF ; ERBB1 ; ERBB2 ; ERBB3 ; ERBB2_3 ; ERBB1_2 ; ERBB1_3			
AKT1_ERalpha	AKT1	0	0	**1 Stable state**
	ERalpha	0	0	
	MEK1 ; CDK2 ; CyclinE1 ; MYC ; EGF ; ERBB1 ; ERBB2 ; ERBB3 ; ERBB2_3 ; ERBB1_2 ; ERBB1_3			
AKT1_MYC	AKT1	0	0	**1 Stable state**

	MYC	0	0	
	MEK1 ; p21 ; p27 ; ERalpha ; EGF ; ERBB1 ; ERBB2 ; ERBB3 ; ERBB2_3 ; ERBB1_2 ; ERBB1_3			
AKT1_CycE1	AKT1	0	0	**1 Stable state**
	CyclinE1	0	0	
	MEK1 ; CDK4 ; CyclinD1 ; **pRB1** ; ERalpha ; CDK6 ; MYC ; EGF ; ERBB1 ; ERBB2 ; ERBB3 ; ERBB2_3 ; ERBB1_2 ; ERBB1_3			
MEK1_CDK2	MEK1	0	0	**1 Stable state**
	CDK2	0	0	
	CDK4 ; CyclinD1 ; CyclinE1 ; **pRB1** ; ERalpha ; AKT1 ; CDK6 ; MYC ; EGF ; ERBB1 ; ERBB2 ; ERBB3 ; ERBB2_3 ; ERBB1_2 ; ERBB1_3			
MEK1_CDK4	MEK1	0	0	**1 Stable state**
	CDK4	0	0	
	CyclinD1 ; CDK2 ; CyclinE1 ; ERalpha ; AKT1 ; CDK6 ; MYC ; EGF ; ERBB1 ; ERBB2 ; ERBB3 ; ERBB2_3 ; ERBB1_2 ; ERBB1_3			
MEK1_p21	MEK1	0	0	**1 Stable state**
	p21	0	0	
	CDK4 ; CyclinD1 ; CDK2 ; CyclinE1 ; **pRB1** ; ERalpha ; AKT1 ; CDK6 ; MYC ; EGF ; ERBB1 ; ERBB2 ; ERBB3 ; ERBB2_3 ; ERBB1_2 ; ERBB1_3			
MEK1_p27	MEK1	0	0	**1 Stable state**
	p27	0	0	
	CDK4 ; CyclinD1 ; CDK2 ; CyclinE1 ; **pRB1** ; ERalpha ; AKT1 ; CDK6 ; MYC ; EGF ; ERBB1 ; ERBB2 ; ERBB3 ; ERBB2_3 ; ERBB1_2 ; ERBB1_3			
MEK1_CycD1	MEK1	0	0	**1 Stable state**
	CyclinD1	0	0	
	CDK2 ; CyclinE1 ; ERalpha ; AKT1 ; MYC ; EGF ; ERBB1 ; ERBB2 ; ERBB3 ; ERBB2_3 ; ERBB1_2 ; ERBB1_3			
MEK1_ERBB2_3	MEK1	0	0	**1 Stable state**
	ERBB2	0	0	
	ERBB3	0	0	
	CDK4 ; CyclinD1 ; CDK2 ; CyclinE1 ; **pRB1** ; ERalpha ; AKT1 ; CDK6 ; MYC ; IGF1R ; EGF ; ERBB1			
MEK1_ERBB1_3	MEK1	0	0	**2 Stable states**
	ERBB1_3	0	0	
	ERBB1	0	0	
	ERBB3	0	0	
	p21 ; p27 ; EGF ; ERBB2			
	CDK4 ; CyclinD1 ; CDK2 ; CyclinE1 ; **pRB1** ; ERalpha ; AKT1 ; CDK6 ; MYC ; IGF1R ; EGF ; ERBB2			
MEK1_ERBB1	MEK1	0	0	**1 Stable state**
	ERBB1	0	0	
	CDK4 ; CyclinD1 ; CDK2 ; CyclinE1 ; **pRB1** ; ERalpha ; AKT1 ; CDK6 ; MYC ; EGF ; ERBB2 ; ERBB3 ; ERBB2_3			
MEK1_ERBB2	MEK1	0	0	**1 Stable state**
	ERBB2	0	0	
	CDK4 ; CyclinD1 ; CDK2 ; CyclinE1 ; **pRB1** ; ERalpha ; AKT1 ; CDK6 ; MYC ; IGF1R ; EGF ; ERBB1 ; ERBB3 ; ERBB1_3			
MEK1_ERBB1_2	MEK1	0	0	**2 Stable states**
	ERBB1_2	0	0	
	ERBB1	0	0	

	ERBB2	0	0	
	p21 ; p27 ; EGF ; ERBB3			
	CDK4 ; CyclinD1 ; CDK2 ; CyclinE1 ; **pRB1** ; ERalpha ; AKT1 ; CDK6 ; MYC ; IGF1R ; EGF ; ERBB3			
MEK1_IGF1R	MEK1	0	0	**1 Stable state**
	IGF1R	0	0	
	CDK4 ; CyclinD1 ; CDK2 ; CyclinE1 ; **pRB1** ; ERalpha ; AKT1 ; CDK6 ; MYC ; EGF ; ERBB1 ; ERBB2 ; ERBB3 ; ERBB2_3 ; ERBB1_2 ; ERBB1_3			
MEK1_CDK6	MEK1	0	0	**1 Stable state**
	CDK6	0	0	
	CDK4 ; CyclinD1 ; CDK2 ; CyclinE1 ; ERalpha ; AKT1 ; MYC ; EGF ; ERBB1 ; ERBB2 ; ERBB3 ; ERBB2_3 ; ERBB1_2 ; ERBB1_3			
MEK1_ERalpha	MEK1	0	0	**1 Stable state**
	ERalpha	0	0	
	CDK2 ; CyclinE1 ; AKT1 ; MYC ; EGF ; ERBB1 ; ERBB2 ; ERBB3 ; ERBB2_3 ; ERBB1_2 ; ERBB1_3			
MEK1_MYC	MEK1	0	0	**1 Stable state**
	MYC	0	0	
	ERalpha ; AKT1 ; EGF ; ERBB1 ; ERBB2 ; ERBB3 ; ERBB2_3 ; ERBB1_2 ; ERBB1_3			
MEK1_CyclE1	MEK1	0	0	**1 Stable state**
	CyclinE1	0	0	
	CDK4 ; CyclinD1 ; **pRB1** ; ERalpha ; AKT1 ; CDK6 ; MYC ; EGF ; ERBB1 ; ERBB2 ; ERBB3 ; ERBB2_3 ; ERBB1_2 ; ERBB1_3			
CDK2_CDK4	CDK2	0	0	**1 Stable state**
	CDK4	0	0	
	MEK1 ; CyclinD1 ; CyclinE1 ; ERalpha ; AKT1 ; CDK6 ; MYC ; EGF ; ERBB1 ; ERBB2 ; ERBB3 ; ERBB2_3 ; ERBB1_2 ; ERBB1_3			
CDK2_p21	CDK2	0	0	**1 Stable state**
	p21	0	0	
	MEK1 ; CDK4 ; CyclinD1 ; CyclinE1 ; **pRB1** ; ERalpha ; AKT1 ; CDK6 ; MYC ; EGF ; ERBB1 ; ERBB2 ; ERBB3 ; ERBB2_3 ; ERBB1_2 ; ERBB1_3			
CDK2_p27	CDK2	0	0	**1 Stable state**
	p27	0	0	
	MEK1 ; CDK4 ; CyclinD1 ; CyclinE1 ; **pRB1** ; ERalpha ; AKT1 ; CDK6 ; MYC ; EGF ; ERBB1 ; ERBB2 ; ERBB3 ; ERBB2_3 ; ERBB1_2 ; ERBB1_3			
CDK2_CycD1	CDK2	0	0	**1 Stable state**
	CyclinD1	0	0	
	MEK1 ; CyclinE1 ; ERalpha ; AKT1 ; MYC ; EGF ; ERBB1 ; ERBB2 ; ERBB3 ; ERBB2_3 ; ERBB1_2 ; ERBB1_3			
CDK2_ERBB2_3	CDK2	0	0	**1 Stable state**
	ERBB2	0	0	
	ERBB3	0	0	
	MEK1 ; CDK4 ; CyclinD1 ; CyclinE1 ; **pRB1** ; ERalpha ; AKT1 ; CDK6 ; MYC ; IGF1R ; EGF ; ERBB1			
CDK2_ERBB1_3	CDK2	0	0	**2 Stable states**
	ERBB1_3	0	0	
	ERBB1	0	0	
	ERBB3	0	0	

	p21 ; p27 ; EGF ; ERBB2			
	MEK1 ; CDK4 ; CyclinD1 ; CyclinE1 ; **pRB1** ; ERalpha ; AKT1 ; CDK6 ; MYC ; IGF1R ; EGF ; ERBB2			
CDK2_ERBB1	CDK2	0	0	**1 Stable state**
	ERBB1	0	0	
	MEK1 ; CDK4 ; CyclinD1 ; CyclinE1 ; **pRB1** ; ERalpha ; AKT1 ; CDK6 ; MYC ; EGF ; ERBB2 ; ERBB3 ; ERBB2_3			
CDK2_ERBB2	CDK2	0	0	**1 Stable state**
	ERBB2	0	0	
	MEK1 ; CDK4 ; CyclinD1 ; CyclinE1 ; **pRB1** ; ERalpha ; AKT1 ; CDK6 ; MYC ; IGF1R ; EGF ; ERBB1 ; ERBB3 ; ERBB1_3			
CDK2_ERBB1_2	CDK2	0	0	**2 Stable states**
	ERBB1	0	0	
	ERBB2	0	0	
	p21 ; p27 ; EGF ; ERBB3			
	MEK1 ; CDK4 ; CyclinD1 ; CyclinE1 ; **pRB1** ; ERalpha ; AKT1 ; CDK6 ; MYC ; IGF1R ; EGF ; ERBB3			
CDK2_IGF1R	CDK2	0	0	**1 Stable state**
	IGF1R	0	0	
	MEK1 ; CDK4 ; CyclinD1 ; CyclinE1 ; **pRB1** ; ERalpha ; AKT1 ; CDK6 ; MYC ; EGF ; ERBB1 ; ERBB2 ; ERBB3 ; ERBB2_3 ; ERBB1_2 ; ERBB1_3			
CDK2_CDK6	CDK2	0	0	**1 Stable state**
	CDK6	0	0	
	MEK1 ; CDK4 ; CyclinD1 ; CyclinE1 ; ERalpha ; AKT1 ; MYC ; EGF ; ERBB1 ; ERBB2 ; ERBB3 ; ERBB2_3 ; ERBB1_2 ; ERBB1_3			
CDK2_ERalpha	CDK2	0	0	**1 Stable state**
	ERalpha	0	0	
	MEK1 ; CyclinE1 ; AKT1 ; MYC ; EGF ; ERBB1 ; ERBB2 ; ERBB3 ; ERBB2_3 ; ERBB1_2 ; ERBB1_3			
CDK2_MYC	CDK2	0	0	**1 Stable state**
	MYC	0	0	
	MEK1 ; ERalpha ; AKT1 ; EGF ; ERBB1 ; ERBB2 ; ERBB3 ; ERBB2_3 ; ERBB1_2 ; ERBB1_3			
CDK2_CycE1	CDK2	0	0	**1 Stable state**
	CyclinE1	0	0	
	MEK1 ; CDK4 ; CyclinD1 ; **pRB1** ; ERalpha ; AKT1 ; CDK6 ; MYC ; EGF ; ERBB1 ; ERBB2 ; ERBB3 ; ERBB2_3 ; ERBB1_2 ; ERBB1_3			
CDK4_p21	CDK4	0	0	**1 Stable state**
	p21	0	0	
	MEK1 ; CyclinD1 ; CDK2 ; CyclinE1 ; ERalpha ; AKT1 ; CDK6 ; MYC ; EGF ; ERBB1 ; ERBB2 ; ERBB3 ; ERBB2_3 ; ERBB1_2 ; ERBB1_3			
CDK4_p27	CDK4	0	0	**1 Stable state**
	p27	0	0	
	MEK1 ; CyclinD1 ; CDK2 ; CyclinE1 ; ERalpha ; AKT1 ; CDK6 ; MYC ; EGF ; ERBB1 ; ERBB2 ; ERBB3 ; ERBB2_3 ; ERBB1_2 ; ERBB1_3			
CDK4_CycD1	CDK4	0	0	**1 Stable state**
	CyclinD1	0	0	
	MEK1 ; CDK2 ; CyclinE1 ; ERalpha ; AKT1 ; MYC ; EGF ; ERBB1 ; ERBB2 ; ERBB3 ; ERBB2_3 ; ERBB1_2 ; ERBB1_3			
CDK4_ERBB2_3	CDK4	0	0	**1 Stable state**

	ERBB2	0	0	
	ERBB3	0	0	
	MEK1 ; CyclinD1 ; CDK2 ; CyclinE1 ; ERalpha ; AKT1 ; CDK6 ; MYC ; IGF1R ; EGF ; ERBB1			
CDK4_ERBB1_3	CDK4	0	0	**2 Stable states**
	ERBB1_3	0	0	
	ERBB1	0	0	
	ERBB3	0	0	
	p21 ; p27 ; EGF ; ERBB2			
	MEK1 ; CyclinD1 ; CDK2 ; CyclinE1 ; ERalpha ; AKT1 ; CDK6 ; MYC ; IGF1R ; EGF ; ERBB2			
CDK4_ERBB1	CDK4	0	0	**1 Stable state**
	ERBB1	0	0	
	MEK1 ; CyclinD1 ; CDK2 ; CyclinE1 ; ERalpha ; AKT1 ; CDK6 ; MYC ; EGF ; ERBB2 ; ERBB3 ; ERBB2_3			
CDK4_ERBB2	CDK4	0	0	**1 Stable state**
	ERBB2	0	0	
	MEK1 ; CyclinD1 ; CDK2 ; CyclinE1 ; ERalpha ; AKT1 ; CDK6 ; MYC ; IGF1R ; EGF ; ERBB1 ; ERBB3 ; ERBB1_3			
CDK4_ERBB1_2	CDK4	0	0	**2 Stable states**
	ERBB1_2	0	0	
	ERBB1	0	0	
	ERBB2	0	0	
	p21 ; p27 ; EGF ; ERBB3			
	MEK1 ; CyclinD1 ; CDK2 ; CyclinE1 ; ERalpha ; AKT1 ; CDK6 ; MYC ; IGF1R ; EGF ; ERBB3			
CDK4_IGF1R	CDK4	0	0	**1 Stable state**
	IGF1R	0	0	
	MEK1 ; CyclinD1 ; CDK2 ; CyclinE1 ; ERalpha ; AKT1 ; CDK6 ; MYC ; EGF ; ERBB1 ; ERBB2 ; ERBB3 ; ERBB2_3 ; ERBB1_2 ; ERBB1_3			
CDK4_CDK6	CDK4	0	0	**1 Stable state**
	CDK6	0	0	
	MEK1 ; CyclinD1 ; CDK2 ; CyclinE1 ; ERalpha ; AKT1 ; MYC ; EGF ; ERBB1 ; ERBB2 ; ERBB3 ; ERBB2_3 ; ERBB1_2 ; ERBB1_3			
CDK4_ERalpha	CDK4	0	0	**1 Stable state**
	ERalpha	0	0	
	MEK1 ; CDK2 ; CyclinE1 ; AKT1 ; MYC ; EGF ; ERBB1 ; ERBB2 ; ERBB3 ; ERBB2_3 ; ERBB1_2 ; ERBB1_3			
CDK4_MYC	CDK4	0	0	**1 Stable state**
	MYC	0	0	
	MEK1 ; ERalpha ; AKT1 ; EGF ; ERBB1 ; ERBB2 ; ERBB3 ; ERBB2_3 ; ERBB1_2 ; ERBB1_3			
CDK4_CycE1	CDK4	0	0	**1 Stable state**
	CyclinE1	0	0	
	MEK1 ; CyclinD1 ; ERalpha ; AKT1 ; CDK6 ; MYC ; EGF ; ERBB1 ; ERBB2 ; ERBB3 ; ERBB2_3 ; ERBB1_2 ; ERBB1_3			
p21_p27	p21	0	0	**1 Stable state**
	p27	0	0	
	MEK1 ; CDK4 ; CyclinD1 ; CDK2 ; CyclinE1 ; **pRB1** ; ERalpha ; AKT1 ; CDK6 ; MYC ; EGF ; ERBB1 ; ERBB2 ; ERBB3 ; ERBB2_3 ; ERBB1_2 ; ERBB1_3			
p21_CycD1	p21	0	0	**1 Stable state**

	CyclinD1	0	0	
	MEK1 ; CDK2 ; CyclinE1 ; ERalpha ; AKT1 ; MYC ; EGF ; ERBB1 ; ERBB2 ; ERBB3 ; ERBB2_3 ; ERBB1_2 ; ERBB1_3			
p21_ERBB2_3	p21	0	0	**1 Stable state**
	ERBB2	0	0	
	ERBB3	0	0	
	MEK1 ; CDK4 ; CyclinD1 ; CDK2 ; CyclinE1 ; **pRB1** ; ERalpha ; AKT1 ; CDK6 ; MYC ; IGF1R ; EGF ; ERBB1			
p21_ERBB1_3	p21	0	0	**2 Stable states**
	ERBB1_3	0	0	
	ERBB1	0	0	
	ERBB3	0	0	
	p27 ; EGF ; ERBB2			
	MEK1 ; CDK4 ; CyclinD1 ; CDK2 ; CyclinE1 ; **pRB1** ; ERalpha ; AKT1 ; CDK6 ; MYC ; IGF1R ; EGF ; ERBB2			
p21_ERBB1	p21	0	0	**1 Stable state**
	ERBB1	0	0	
	MEK1 ; CDK4 ; CyclinD1 ; CDK2 ; CyclinE1 ; **pRB1** ; ERalpha ; AKT1 ; CDK6 ; MYC ; EGF ; ERBB2 ; ERBB3 ; ERBB2_3			
p21_ERBB2	p21	0	0	**1 Stable state**
	ERBB2	0	0	
	MEK1 ; CDK4 ; CyclinD1 ; CDK2 ; CyclinE1 ; **pRB1** ; ERalpha ; AKT1 ; CDK6 ; MYC ; IGF1R ; EGF ; ERBB1 ; ERBB3 ; ERBB1_3			
p21_ERBB1_2	p21	0	0	**2 Stable states**
	ERBB2	0	0	
	ERBB1_2	0	0	
	ERBB1	0	0	
	p27 ; EGF ; ERBB3			
	MEK1 ; CDK4 ; CyclinD1 ; CDK2 ; CyclinE1 ; **pRB1** ; ERalpha ; AKT1 ; CDK6 ; MYC ; IGF1R ; EGF ; ERBB3			
p21_IGF1R	p21	0	0	**1 Stable state**
	IGF1R	0	0	
	MEK1 ; CDK4 ; CyclinD1 ; CDK2 ; CyclinE1 ; **pRB1** ; ERalpha ; AKT1 ; CDK6 ; MYC ; EGF ; ERBB1 ; ERBB2 ; ERBB3 ; ERBB2_3 ; ERBB1_2 ; ERBB1_3			
p21_CDK6	p21	0	0	**1 Stable state**
	CDK6	0	0	
	MEK1 ; CDK4 ; CyclinD1 ; CDK2 ; CyclinE1 ; ERalpha ; AKT1 ; MYC ; EGF ; ERBB1 ; ERBB2 ; ERBB3 ; ERBB2_3 ; ERBB1_2 ; ERBB1_3			
p21_ERalpha	p21	0	0	**1 Stable state**
	ERalpha	0	0	
	MEK1 ; CDK2 ; CyclinE1 ; AKT1 ; MYC ; EGF ; ERBB1 ; ERBB2 ; ERBB3 ; ERBB2_3 ; ERBB1_2 ; ERBB1_3			
p21_MYC	p21	0	0	**1 Stable state**
	MYC	0	0	
	MEK1 ; ERalpha ; AKT1 ; EGF ; ERBB1 ; ERBB2 ; ERBB3 ; ERBB2_3 ; ERBB1_2 ; ERBB1_3			
p21_CycE1	p21	0	0	**1 Stable state**
	CyclinE1	0	0	
	MEK1 ; CDK4 ; CyclinD1 ; **pRB1** ; ERalpha ; AKT1 ; CDK6 ; MYC ; EGF ; ERBB1 ; ERBB2 ; ERBB3 ; ERBB2_3 ; ERBB1_2 ; ERBB1_3			
p27_CycD1	p27	0	0	**1 Stable state**

	CyclinD1	0	0	
	MEK1 ; CDK2 ; CyclinE1 ; ERalpha ; AKT1 ; MYC ; EGF ; ERBB1 ; ERBB2 ; ERBB3 ; ERBB2_3 ; ERBB1_2 ; ERBB1_3			
p27_ERBB2_3	p27	0	0	**1 Stable state**
	ERBB2	0	0	
	ERBB3	0	0	
	MEK1 ; CDK4 ; CyclinD1 ; CDK2 ; CyclinE1 ; **pRB1** ; ERalpha ; AKT1 ; CDK6 ; MYC ; IGF1R ; EGF ; ERBB1			
p27_ERBB1_3	p27	0	0	**2 Stable states**
	ERBB1_3	0	0	
	ERBB1	0	0	
	ERBB3	0	0	
	p21 ; EGF ; ERBB2			
	MEK1 ; CDK4 ; CyclinD1 ; CDK2 ; CyclinE1 ; **pRB1** ; ERalpha ; AKT1 ; CDK6 ; MYC ; IGF1R ; EGF ; ERBB2			
p27_ERBB1	p27	0	0	**1 Stable state**
	ERBB1	0	0	
	MEK1 ; CDK4 ; CyclinD1 ; CDK2 ; CyclinE1 ; **pRB1** ; ERalpha ; AKT1 ; CDK6 ; MYC ; EGF ; ERBB2 ; ERBB3 ; ERBB2_3			
p27_ERBB2	p27	0	0	**1 Stable state**
	ERBB2	0	0	
	MEK1 ; CDK4 ; CyclinD1 ; CDK2 ; CyclinE1 ; **pRB1** ; ERalpha ; AKT1 ; CDK6 ; MYC ; IGF1R ; EGF ; ERBB1 ; ERBB3 ; ERBB1_3			
p27_ERBB1_2	p27	0	0	**2 Stable states**
	ERBB2	0	0	
	ERBB1_2	0	0	
	ERBB1	0	0	
	p21 ; EGF ; ERBB3			
	MEK1 ; CDK4 ; CyclinD1 ; CDK2 ; CyclinE1 ; **pRB1** ; ERalpha ; AKT1 ; CDK6 ; MYC ; IGF1R ; EGF ; ERBB3			
p27_IGF1R	p27	0	0	**1 Stable state**
	IGF1R	0	0	
	MEK1 ; CDK4 ; CyclinD1 ; CDK2 ; CyclinE1 ; **pRB1** ; ERalpha ; AKT1 ; CDK6 ; MYC ; EGF ; ERBB1 ; ERBB2 ; ERBB3 ; ERBB2_3 ; ERBB1_2 ; ERBB1_3			
p27_CDK6	p27	0	0	**1 Stable state**
	CDK6	0	0	
	MEK1 ; CDK4 ; CyclinD1 ; CDK2 ; CyclinE1 ; ERalpha ; AKT1 ; MYC ; EGF ; ERBB1 ; ERBB2 ; ERBB3 ; ERBB2_3 ; ERBB1_2 ; ERBB1_3			
p27_ERalpha	p27	0	0	**1 Stable state**
	ERalpha	0	0	
	MEK1 ; CDK2 ; CyclinE1 ; AKT1 ; MYC ; EGF ; ERBB1 ; ERBB2 ; ERBB3 ; ERBB2_3 ; ERBB1_2 ; ERBB1_3			
p27_MYC	p27	0	0	**1 Stable state**
	MYC	0	0	
	MEK1 ; ERalpha ; AKT1 ; EGF ; ERBB1 ; ERBB2 ; ERBB3 ; ERBB2_3 ; ERBB1_2 ; ERBB1_3			
p27_CycE1	p27	0	0	**1 Stable state**
	CyclinE1	0	0	
	MEK1 ; CDK4 ; CyclinD1 ; **pRB1** ; ERalpha ; AKT1 ; CDK6 ; MYC ; EGF ; ERBB1 ; ERBB2 ; ERBB3 ; ERBB2_3 ; ERBB1_2 ; ERBB1_3			
CycD1_ERBB2_3	CyclinD1	0	0	**1 Stable state**

	ERBB2	0	0	
	ERBB3	0	0	
	MEK1 ; CDK2 ; CyclinE1 ; ERalpha ; AKT1 ; MYC ; IGF1R ; EGF ; ERBB1			
CycD1_ERBB1_3	CyclinD1	0	0	**2 Stable states**
	ERBB1_3	0	0	
	ERBB1	0	0	
	ERBB3	0	0	
	p21 ; p27 ; EGF ; ERBB2			
	MEK1 ; CDK2 ; CyclinE1 ; ERalpha ; AKT1 ; MYC ; IGF1R ; EGF ; ERBB2			
CycD1_ERBB1	CyclinD1	0	0	**1 Stable state**
	ERBB1	0	0	
	MEK1 ; CDK2 ; CyclinE1 ; ERalpha ; AKT1 ; MYC ; EGF ; ERBB2 ; ERBB3 ; ERBB2_3			
CycD1_ERBB2	CyclinD1	0	0	**1 Stable state**
	ERBB2	0	0	
	MEK1 ; CDK2 ; CyclinE1 ; ERalpha ; AKT1 ; MYC ; IGF1R ; EGF ; ERBB1 ; ERBB3 ; ERBB1_3			
CycD1_ERBB1_2	CyclinD1	0	0	**2 Stable states**
	ERBB2	0	0	
	ERBB1_2	0	0	
	ERBB1	0	0	
	p21 ; p27 ; EGF ; ERBB3			
	MEK1 ; CDK2 ; CyclinE1 ; ERalpha ; AKT1 ; MYC ; IGF1R ; EGF ; ERBB3			
CycD1_IGF1R	CyclinD1	0	0	**1 Stable state**
	IGF1R	0	0	
	MEK1 ; CDK2 ; CyclinE1 ; ERalpha ; AKT1 ; MYC ; EGF ; ERBB1 ; ERBB2 ; ERBB3 ; ERBB2_3 ; ERBB1_2 ; ERBB1_3			
CycD1_CDK6	CyclinD1	0	0	**1 Stable state**
	CDK6	0	0	
	MEK1 ; CDK2 ; CyclinE1 ; ERalpha ; AKT1 ; MYC ; EGF ; ERBB1 ; ERBB2 ; ERBB3 ; ERBB2_3 ; ERBB1_2 ; ERBB1_3			
CycD1_ERalpha	CyclinD1	0	0	**1 Stable state**
	ERalpha	0	0	
	MEK1 ; CDK2 ; CyclinE1 ; AKT1 ; MYC ; EGF ; ERBB1 ; ERBB2 ; ERBB3 ; ERBB2_3 ; ERBB1_2 ; ERBB1_3			
CycD1_MYC	CyclinD1	0	0	**1 Stable state**
	MYC	0	0	
	MEK1 ; ERalpha ; AKT1 ; EGF ; ERBB1 ; ERBB2 ; ERBB3 ; ERBB2_3 ; ERBB1_2 ; ERBB1_3			
CycD1_CycE1	CyclinD1	0	0	**1 Stable state**
	CyclinE1	0	0	
	MEK1 ; ERalpha ; AKT1 ; MYC ; EGF ; ERBB1 ; ERBB2 ; ERBB3 ; ERBB2_3 ; ERBB1_2 ; ERBB1_3			
ERBB1_2_3	ERBB2	0	0	**2 Stable states**
	ERBB3	0	0	
	ERBB1_2	0	0	
	ERBB1_3	0	0	
	ERBB1	0	0	
	p21 ; p27 ; EGF			
	MEK1 ; CDK4 ; CyclinD1 ; CDK2 ; CyclinE1 ; **pRB1** ; ERalpha ; AKT1 ; CDK6 ; MYC ; IGF1R ; EGF			

ERBB2_3_IGFR1	ERBB2	0	0	**1 Stable state**
	ERBB3	0	0	
	IGF1R	0	0	
	MEK1 ; CDK4 ; CyclinD1 ; CDK2 ; CyclinE1 ; **pRB1** ; ERalpha ; AKT1 ; CDK6 ; MYC ; EGF ; ERBB1			
ERBB2_3_CDK6	ERBB2	0	0	**1 Stable state**
	ERBB3	0	0	
	CDK6	0	0	
	MEK1 ; CDK4 ; CyclinD1 ; CDK2 ; CyclinE1 ; ERalpha ; AKT1 ; MYC ; IGF1R ; EGF ; ERBB1			
ERBB2_3_ERalpha	ERBB2	0	0	**1 Stable state**
	ERBB3	0	0	
	ERalpha	0	0	
	MEK1 ; CDK2 ; CyclinE1 ; AKT1 ; MYC ; IGF1R ; EGF ; ERBB1			
ERBB2_3_MYC	ERBB2	0	0	**1 Stable state**
	ERBB3	0	0	
	MYC	0	0	
	MEK1 ; ERalpha ; AKT1 ; IGF1R ; EGF ; ERBB1			
ERBB2_3_CycE1	ERBB2	0	0	**1 Stable state**
	ERBB3	0	0	
	CyclinE1	0	0	
	MEK1 ; CDK4 ; CyclinD1 ; **pRB1** ; ERalpha ; AKT1 ; CDK6 ; MYC ; IGF1R ; EGF ; ERBB1			
ERBB1_3_IGF1R	ERBB1_3	0	0	**1 Stable state**
	ERBB1	0	0	
	ERBB3	0	0	
	IGF1R	0	0	
	p21 ; p27 ; EGF ; ERBB2			
ERBB1_3_CDK6	ERBB1_3	0	0	**2 Stable states**
	ERBB1	0	0	
	ERBB3	0	0	
	CDK6	0	0	
	p21 ; p27 ; EGF ; ERBB2			
	MEK1 ; CDK4 ; CyclinD1 ; CDK2 ; CyclinE1 ; ERalpha ; AKT1 ; MYC ; IGF1R ; EGF ; ERBB2			
ERBB1_3_ERalpha	ERBB1_3	0	0	**2 Stable states**
	ERBB1	0	0	
	ERBB3	0	0	
	ERalpha	0	0	
	p21 ; p27 ; EGF ; ERBB2			
	MEK1 ; CDK2 ; CyclinE1 ; AKT1 ; MYC ; IGF1R ; EGF ; ERBB2			
ERBB1_3_MYC	ERBB1_3	0	0	**2 Stable states**
	ERBB1	0	0	
	ERBB3	0	0	
	MYC	0	0	
	p21 ; p27 ; EGF ; ERBB2			
	MEK1 ; ERalpha ; AKT1 ; IGF1R ; EGF ; ERBB2			

ERBB1_3_CycE1	ERBB1_3	0	0	**2 Stable states**
	ERBB1	0	0	
	ERBB3	0	0	
	CyclinE1	0	0	
	p21 ; p27 ; EGF ; ERBB2			
	MEK1 ; CDK4 ; CyclinD1 ; **pRB1** ; ERalpha ; AKT1 ; CDK6 ; MYC ; IGF1R ; EGF ; ERBB2			
ERBB1_IGF1R	ERBB1	0	0	**1 Stable state**
	IGF1R	0	0	
	MEK1 ; CDK4 ; CyclinD1 ; CDK2 ; CyclinE1 ; **pRB1** ; ERalpha ; AKT1 ; CDK6 ; MYC ; EGF ; ERBB2 ; ERBB3 ; ERBB2_3			
ERBB1_CDK6	ERBB1	0	0	**1 Stable state**
	CDK6	0	0	
	MEK1 ; CDK4 ; CyclinD1 ; CDK2 ; CyclinE1 ; ERalpha ; AKT1 ; MYC ; EGF ; ERBB2 ; ERBB3 ; ERBB2_3			
ERBB1_ERalpha	ERBB1	0	0	**1 Stable state**
	ERalpha	0	0	
	MEK1 ; CDK2 ; CyclinE1 ; AKT1 ; MYC ; EGF ; ERBB2 ; ERBB3 ; ERBB2_3			
ERBB1_MYC	ERBB1	0	0	**1 Stable state**
	MYC	0	0	
	MEK1 ; ERalpha ; AKT1 ; EGF ; ERBB2 ; ERBB3 ; ERBB2_3			
ERBB1_CycE1	ERBB1	0	0	**1 Stable state**
	CyclinE1	0	0	
	MEK1 ; CDK4 ; CyclinD1 ; **pRB1** ; ERalpha ; AKT1 ; CDK6 ; MYC ; EGF ; ERBB2 ; ERBB3 ; ERBB2_3			
IGF1R_CDK6	IGF1R	0	0	**1 Stable state**
	CDK6	0	0	
	MEK1 ; CDK4 ; CyclinD1 ; CDK2 ; CyclinE1 ; ERalpha ; AKT1 ; MYC ; EGF ; ERBB1 ; ERBB2 ; ERBB3 ; ERBB2_3 ; ERBB1_2 ; ERBB1_3			
IGF1R_ERalpha	IGF1R	0	0	**1 Stable state**
	ERalpha	0	0	
	MEK1 ; CDK2 ; CyclinE1 ; AKT1 ; MYC ; EGF ; ERBB1 ; ERBB2 ; ERBB3 ; ERBB2_3 ; ERBB1_2 ; ERBB1_3			
IGF1R_MYC	IGF1R	0	0	**1 Stable state**
	MYC	0	0	
	MEK1 ; ERalpha ; AKT1 ; EGF ; ERBB1 ; ERBB2 ; ERBB3 ; ERBB2_3 ; ERBB1_2 ; ERBB1_3			
IGF1R_CycE1	IGF1R	0	0	**1 Stable state**
	CyclinE1	0	0	
	MEK1 ; CDK4 ; CyclinD1 ; **pRB1** ; ERalpha ; AKT1 ; CDK6 ; MYC ; EGF ; ERBB1 ; ERBB2 ; ERBB3 ; ERBB2_3 ; ERBB1_2 ; ERBB1_3			
CDK6_ERalpha	CDK6	0	0	**1 Stable state**
	ERalpha	0	0	
	MEK1 ; CDK2 ; CyclinE1 ; AKT1 ; MYC ; EGF ; ERBB1 ; ERBB2 ; ERBB3 ; ERBB2_3 ; ERBB1_2 ; ERBB1_3			
CDK6_MYC	CDK6	0	0	**1 Stable state**
	MYC	0	0	
	MEK1 ; ERalpha ; AKT1 ; EGF ; ERBB1 ; ERBB2 ; ERBB3 ; ERBB2_3 ; ERBB1_2 ; ERBB1_3			
CDK6_CycE1	CDK6	0	0	**1 Stable state**
	CyclinE1	0	0	

	MEK1 ; CDK4 ; CyclinD1 ; ERalpha ; AKT1 ; MYC ; EGF ; ERBB1 ; ERBB2 ; ERBB3 ; ERBB2_3 ; ERBB1_2 ; ERBB1_3			
ERalpha_MYC	ERalpha	0	0	**1 Stable state**
	MYC	0	0	
	MEK1 ; AKT1 ; EGF ; ERBB1 ; ERBB2 ; ERBB3 ; ERBB2_3 ; ERBB1_2 ; ERBB1_3			
ERalpha_CyclinE1	ERalpha	0	0	**1 Stable state**
	CyclinE1	0	0	
	MEK1 ; AKT1 ; MYC ; EGF ; ERBB1 ; ERBB2 ; ERBB3 ; ERBB2_3 ; ERBB1_2 ; ERBB1_3			
MYC_CycE1	CyclinE1	0	0	**1 Stable state**
	ERalpha	0	0	
	MEK1 ; AKT1 ; MYC ; EGF ; ERBB1 ; ERBB2 ; ERBB3 ; ERBB2_3 ; ERBB1_2 ; ERBB1_3			

Table 2 Analysis of the effects of knockdowns on G1/S transition (p-pRB response).

	**ERBB1_2_3**	**ERalpha**	**AKT1**	**ERalpha_AKT1**
**P-value**	<0.001	<0.001	<0.001	<0.001
**Similarity to MOCK**	Yes	No	Yes	No
**Boolean interpretation (pRB response)**	1	0	1	0
